# Supplementary Respiratory Therapy Improves Pulmonary Function in Pediatric Patients with Cerebral Palsy: A Systematic Review and Meta-Analysis

**DOI:** 10.3390/jcm13030888

**Published:** 2024-02-02

**Authors:** Erika Kolumbán, Márton Szabados, Márk Hernádfői, Uyen Nguyen Do To, Rita Nagy, Ádám Zolcsák, Katalin Eszter Müller, Zoltán Sipos, Dániel Sándor Veres, Anett Szőllősi, Péter Hegyi, Miklós Garami, Ibolya Túri

**Affiliations:** 1 Centre for Translational Medicine, Semmelweis University, 1094 Budapest, Hungaryhernadfoi.mark.viktor@semmelweis.hu (M.H.); nagy.rita3@semmelweis-univ.hu (R.N.); zolcsak.adam@semmelweis.hu (Á.Z.); veres.daniel@med.semmelweis-univ.hu (D.S.V.); hegyi.peter@semmelweis.hu (P.H.); garami.miklos@semmelweis.hu (M.G.); turi.ibolya@semmelweis.hu (I.T.); 2 András Pető Faculty, Semmelweis University, 1125 Budapest, Hungary; uyen.nguyen97@stud.semmelweis.hu; 3 Pediatric Center, Semmelweis University, 1083 Budapest, Hungary; 4 Bethesda Children’s Hospital, 1146 Budapest, Hungary; szollosi.anett@bethesda.hu; 5 Heim Pál National Pediatric Institute, 1089 Budapest, Hungary; katalin.muller@semmelweis.hu; 6 Institute for Translational Medicine, Medical School, University of Pécs, 7624 Pécs, Hungary; sizfab.t.jpte@pte.hu; 7 Department of Biophysics and Radiation Biology, Semmelweis University, 1089 Budapest, Hungary; 8 Department of Family Care Methodology, Faculty of Health Sciences, Semmelweis University, 1088 Budapest, Hungary; 9 Institute of Bioanalysis, Medical School, University of Pécs, 7624 Pécs, Hungary; 10 Institute of Pancreatic Diseases, Semmelweis University, 1085 Budapest, Hungary

**Keywords:** cerebral palsy, supplementary respiratory therapy, pulmonary function, respiratory muscle strength, quality of life

## Abstract

Background: Despite medical advances, individuals with cerebral palsy (CP) face significant respiratory challenges, leading to heightened hospitalization rates and early mortality among this population. We hypothesize that integrating supplementary respiratory therapy into standard rehabilitation will result in significant improvements in pulmonary function, enhanced respiratory muscle strength, and an overall increase in the quality of life among pediatric patients with CP. Methods: A systematic search of literature across five databases was conducted, and random-effects meta-analyses were performed to assess the impact of supplementary respiratory therapy on (a) pulmonary function: forced vital capacity (FVC), forced expiratory volume in 1 s (FEV1), FVC/FEV1 ratio, peak expiratory flow (PEF), and (b) respiratory muscle strength: maximal inspiratory and expiratory pressure (MIP, MEP), and (c) quality of life. Certainty of evidence was determined by the GRADE assessment. Results: Analysis of data from 11 eligible randomized controlled trials revealed clinically meaningful changes in pulmonary function. We found a relevant mean difference (MD) in absolute PEF of 0.50 L/s (95% confidence interval (CI): 0.19; 0.82 *p* = 0.0107). The certainty of the evidence ranged from moderate to high. Conclusions: This study presents current evidence on the impact of various supplementary respiratory therapies for CP patients classified under gross motor function classification level I–IV, demonstrating clinically meaningful improvements in pulmonary function and respiratory muscle strength. These improvements suggest the potential for an enhanced quality of life. Our findings hold the promise of serving as a foundational reference for potential revisions to conventional rehabilitation care, incorporating supplementary respiratory therapy.

## 1. Introduction

Despite significant advancements in medical and rehabilitation treatments, individuals with cerebral palsy (CP) continue to face substantial challenges, particularly in terms of respiratory health [1,2]. These multifaceted challenges include oropharyngeal dysfunction leading to recurrent aspiration, gastro-esophageal reflux, airway obstruction, hypoventilation, spine and chest wall, and frequent respiratory infections [3,4]. Emerging evidence suggests that, although brain damage may not directly cause respiratory issues, children with CP have significantly lower values of respiratory status than typically developing children. Adults with CP have a 14-fold increased risk of death from respiratory system diseases than the general population [5]. Respiratory problems are the leading cause of hospital admission and premature death among people with CP, especially those with GMFCS V, whose complications are predictable and partially preventable [4,6,7,8,9].

Furthermore, Wang et al. have highlighted the fact that respiratory muscle strength is closely associated with self-care activities and social adaptiveness in daily life [10]. This suggests that enhancing respiratory status could significantly improve both quality of life and life expectancy. Despite the high mortality due to pulmonary failure, supplementary respiratory therapy is not a standard treatment element, leading to its varied application globally. The current CP management consensus statement recommends regular preventive exercises and assessments but lacks detailed protocols for incorporating supplementary respiratory therapy into standard care [11]. This lack of guidance might be a factor in the unchanging respiratory health and mortality rates in the CP population.

Supplementary respiratory therapy in the context of caring for children with CP refers to additional respiratory exercises and techniques that complement standard rehabilitation. These therapies include various methods such as breathing exercises, respiratory muscle training, and the use of incentive spirometers. Their significance lies in addressing respiratory challenges faced by children with CP, such as muscle weakness and poor co-ordination, which can lead to serious complications such as pneumonia. The purpose of these therapies is to improve respiratory muscle strength, co-ordination, lung function, and overall quality of life, thereby enhancing the effectiveness of coughing, clearing secretions, and reducing the risk of respiratory infections. The proposed supplementary therapies are accessible, cost-effective, and have no known side effects, making them a viable option for all CP patients.

A recent meta-analysis found that integrating respiratory therapy with standard rehabilitation care significantly enhances pulmonary function and respiratory strength [12]. However, the ideal supplementary therapy and its long-term effects are still not clear. This study’s strength lies in its updated meta-analysis, which includes newer articles and a more statistically sophisticated examination of results, providing a more thorough analysis.

Consequently, a systematic review and meta-analysis were performed to evaluate the effects of supplementary respiratory therapy on respiratory health, quality of life, and overall survival in children with CP.

## 2. Methods

### 2.1. Study Design

The guidelines of preferred reporting items for systematic reviews and meta-analyses (PRISMA 2020) and the suggestions of the Cochrane Handbook were followed [13,14]. Our study was registered under the PROSPERO registration number CRD42022379780.

### 2.2. Information Sources

The final systematic search was conducted in five databases: Medline (via PubMed), Embase, Cochrane Central Register of Controlled Trials (Central), Scopus, and Web of Science on 23 November 2022. Citationchaser was used to identify relevant studies from the reference lists of the eligible articles and citing articles [15].

### 2.3. Eligibility Criteria

The analysis used the patient, intervention, comparison intervention, and outcome (PICO) model, focusing on comparing the effects of conventional therapy alone versus conventional care supplemented with respiratory therapy in pediatric patients with CP [16]. The term “conventional therapy” refers to standard rehabilitation care, identified, and acknowledged by healthcare providers as an established approach to the management of CP. 

This review focused on randomized controlled trials. Studies that included participants with other CP-related cardiac and pulmonary conditions were excluded from the analysis. The primary outcomes of interest were pulmonary function tests (PFTs) such as forced vital capacity (FVC), forced expiratory volume in one second (FEV1), FVC and FEV1 ratio (FEV1/FVC), peak expiratory flow (PEF), respiratory muscle strength measures such as maximum inspiratory pressure (MIP) and maximum expiratory pressure (MEP) and quality of life (QoL) measures.

### 2.4. Search Strategy

Our search key encompassed two domains related to CP and respiratory therapy (Table S2). We did not impose any restrictions on the type of publications to be included, nor did we limit our search by language or other criteria.

### 2.5. Selection Process

The articles retrieved through our search query were imported into reference management software, specifically EndNote 20, by Clarivate Analytics based in Philadelphia, PA, USA, and Rayyan QCRI. Duplicates were automatically and manually removed using the Rayyan QCRI reference management tool. After duplicate removal, articles were screened by two independent authors (EK, MSZ). Disagreements between reviewers were resolved by consensus or by the decision of a third independent reviewer (MH). Cohen’s kappa coefficient was calculated after each phase.

### 2.6. Data Collection Form

Data extraction from eligible articles was performed by three authors independently (KE, QL, MSZ) using a data collection form in Microsoft Excel (Microsoft Corp. Microsoft Excel 2019, Redmond, WA, USA). Any discrepancies or inquiries during the data extraction process were resolved through discussions between the authors.

### 2.7. Risk of Bias Assessment

Two reviewers (EK and MSZ) independently evaluated the risk of bias in the studies using the Rob2 tool (Version 22 August 2019). Any disagreements were resolved by a third investigator (MH).

### 2.8. Certainty of Evidence

The Grading of Recommendations Assessment, Development, and Evaluation (GRADE) approach was used to evaluate the quality of evidence for the outcomes in our clinical question. We used GRADEpro GDT 2015 (software; McMaster University and Evidence Prime, 2022, available from gradepro.org) to interpret the results [17].

### 2.9. Statistical Synthesis

As we assumed considerable between-study heterogeneity due to the nature of clinical studies in this field, we used random-effects models to pool the effect sizes in our meta-analyses. The difference between the means (MD) was used for the effect size measure with a 95% confidence interval (CI) for each outcome. To calculate the study MDs and pooled MDs, the sample size, mean, and corresponding standard deviation (SD) were extracted from each study (separately for each group). Respiratory parameters could be reported in two ways: absolute values (as FVC in L or FEV1 in L/1s) or relative as a percentage of the value predicted based on gender, age, ethnicity, height, and weight to the corresponding mean value at the individual level for a given age and sex. These measures were pooled separately (most of the studies reported only one, and a few reported both). We reported the results as experimental group minus control group values. We performed analyses for both post-intervention (given as main results), and pre-intervention values, and the change during the intervention. We implemented two models to analyze the change values. The first was based only on the articles where the mean and SD were given-referred to as direct change in the text. In the second model, we imputed a correlation coefficient and estimated the change for each article. As the latter needs an assumption for the correlation coefficient between post- and pre-treatment values, we reported these results in the text as estimated change.

Results were considered statistically significant if the pooled CI did not contain the null value. We summarized the findings related to the meta-analysis on forest plots. Where applicable—when the study number was large enough and not too heterogeneous—we also reported the prediction intervals (i.e., the expected range of effects of future studies) of the results. Additionally, between-study heterogeneity was described by Higgins and Thompson’s I^2^ statistics [18]. Small-study publication bias was assessed by visual inspection of Funnel plots and calculating classical Egger’s test *p*-value [19]. We hypothesized possible small study bias if the *p*-value was less than 10%. (However, we kept in mind that the test had limited diagnostic assessment below ~10 studies.) Potential outlier publications were explored using different influence measures and plots following the recommendations of Harrer et al. [20]. Additionally, we performed multivariate analyses of variables that might show high correlation: FVC-FEV1 and MIP-MEP values. 

All statistical analyses were made with R software (R Core Team 2023, v4.2.3) using the meta (Schwarzer 2023, v6.2.1) package for basic meta-analysis calculations and plots, and the dmetar (Cuijpers, Furukawa, and Ebert 2023, v0.0.9000) package for additional influential analysis calculations and plots. For multilevel and multivariate models, the metafor package (Viechtbauer 2023, v4.0.0) was also used.

Results are presented in the format as MD [95% CI lower limit; upper limit]. Additional details on calculations, data synthesis, publication bias assessment, and influential analyses are available in the Supplementary Material.

### 2.10. Estimation of Minimal Clinically Relevant Effect

A 5% increment after the intervention was defined as the clinically minimally relevant effect, calculated by taking the mean of the mean values reported in the studies and finding 5% of that mean.

## 3. Results

### 3.1. Search and Selection

**Figure 1:**
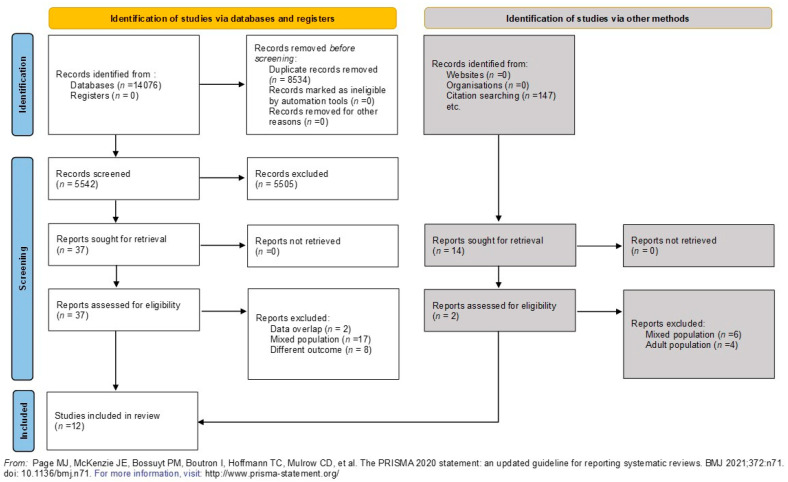
PRISMA flow diagram tool chart illustrating the selection process [13].

The search in the databases yielded 14,076 records that initially seemed relevant. After discarding 8534 duplicates, 5542 titles and abstracts were reviewed. Of these, 5505 were not suitable based on the eligibility criteria, leaving 37 studies for full-text examination. Eventually, 12 studies involving 446 patients were selected for the final analysis, all of which met the inclusion and exclusion criteria. Out of these, 11 studies were incorporated into the meta-analyses. The entire selection process is depicted in the PRISMA flowchart (Figure 1).

### 3.2. Basic Characteristics of Studies Included

The baseline characteristics of the studies included are detailed in Table S1. All 12 articles were randomized controlled trials and six distinct approaches were identified that could be categorized as supplementary respiratory therapy, namely: manual diaphragmatic stretching technique [21], incentive spirometer [22,23] inspiratory muscle training [24,25,26], breathing exercise series [25,26], feedback respiratory training [27,28] and upper extremity resistance exercise with elastic bands [29,30]. We considered the different supplementary respiratory therapies as a single category. Different countries use various terminologies such as conventional, standard, or traditional to describe the typical therapy or standard care provided to patients with CP. We treated these varied terminologies as a unified entity, serving as a common comparator for our study. All studies employed spirometry to measure pulmonary functions.

### 3.3. Pulmonary Function Tests Demonstrate Clinically Relevant Improvement following the Intervention

#### 3.3.1. Change in FVC Values Due to the Intervention

Ten studies reported data on FVC using liters or percentages as measurement units.

**Figure 2:**
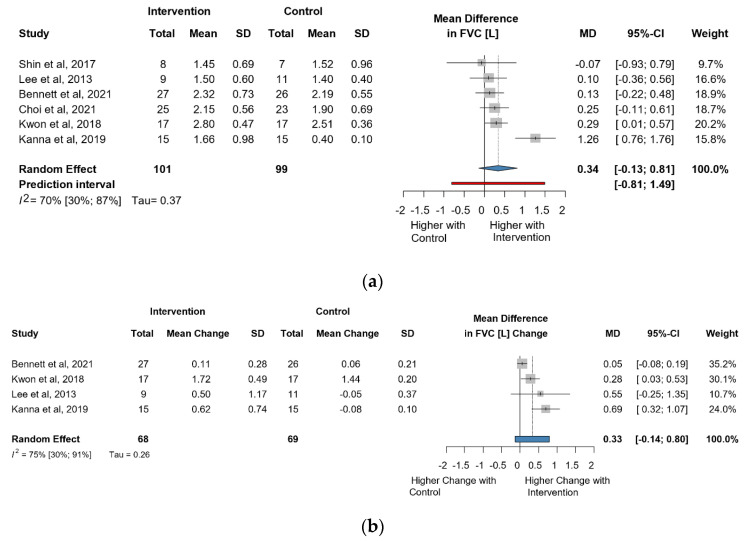
(**a**). Forest plot of FVC values, in liters, of cerebral palsy patients after supplementary respiratory therapy plus conventional care versus conventional care. (**b**). Forest plot of direct change in FVC values, in liters, of cerebral palsy patients after supplementary respiratory therapy plus conventional care versus conventional care [21,22,24,26,29,31].

The meta-analysis of six studies and 200 observations assessed the impact of the intervention on FVC in liters and showed a statistically non-significant increase in FVC(L) (MD = 0.34 [(−)0.13; 0.81], *p* = 0.1325) (Figure 2a). Similarly, the evaluation of the direct change after the intervention in 137 observations showed a statistically non-significant change in FVC(L) MD = 0.33 [(−)0.13; 0.79] *p* = 0.1102) (Figure 2b). The estimated change in FVC(L) did not result in a significant change either (MD = 0.19 [(−)0.18; 0.57] *p* = 0.2504) (Figure S4).

**Figure 3:**
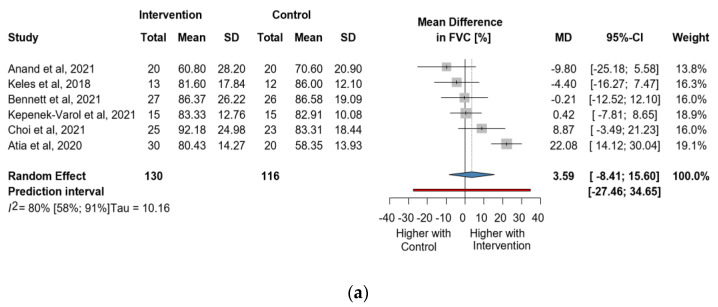

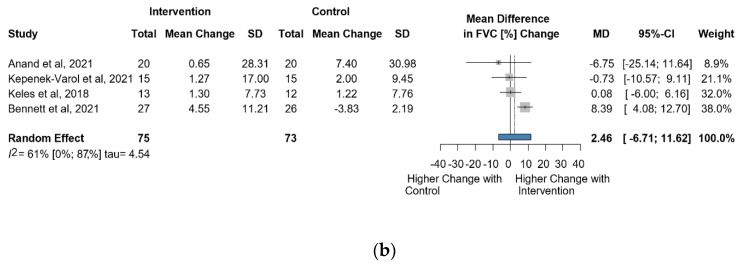
(**a**). Forest plot of FVC values in % of cerebral palsy patients after supplementary respiratory therapy plus conventional care versus conventional care. (**b**). Forest plot of direct change in FVC values in % of cerebral palsy patients after supplementary respiratory therapy plus conventional care versus conventional care [21,22,23,31,32,33].

Six studies and 246 observations showed a statistically non-significant change in FVC (%) MD = 3.59 [(−)8.41; 15.60], *p* = 0.476) (Figure 3a). Similarly, the direct change for FVC (%), in 148 observations analyzed estimated a statistically non-significant change (MD = 2.46 [(−)6.71; 11.6] *p* = 0.4656) (Figure 3b). The estimated change in FVC (%) did not show a statistically significant effect (MD = (−)4.5134 [(−)15.52; 6.49] *p* = 0.3404) (Figure S5).

#### 3.3.2. Change in FEV1 Due to Intervention

Seven studies reported data on FEV1 using liters or percentages as measurement units.

**Figure 4:**
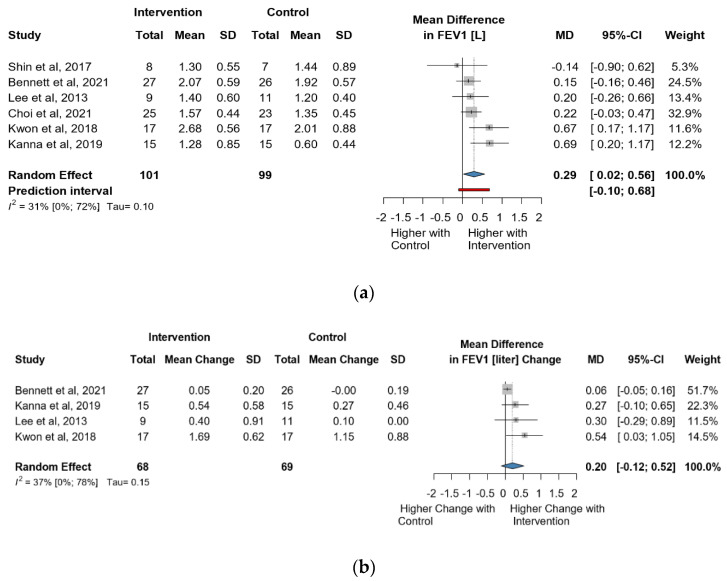
(**a**). Forest plot of FEV1 values, in liters, of cerebral palsy patients after supplementary respiratory therapy plus conventional care versus conventional care. (**b**). Forest plot of direct change in FEV1 values, in liters, of cerebral palsy patients after supplementary respiratory therapy plus conventional care versus conventional care [21,24,26,30].

Six studies and 200 observations assessed the impact of the intervention on FEV1(L) and showed a statistically significant increase (MD = 0.29 [0.02; 0.55], *p* = 0.0374), (Figure 4a) and the direct change analyzed in 137 observations indicated a statistically non-significant change in FEV1(L) (MD = 0.20 [(−)0.12; 0.52] *p* = 0.1367) (Figure 4b). The estimated change in FEV1(L) showed no statistically significant effect (MD = 0.10 [(−)0.15; 0.35] *p* = 0.3517) (Figure S6).

**Figure 5:**
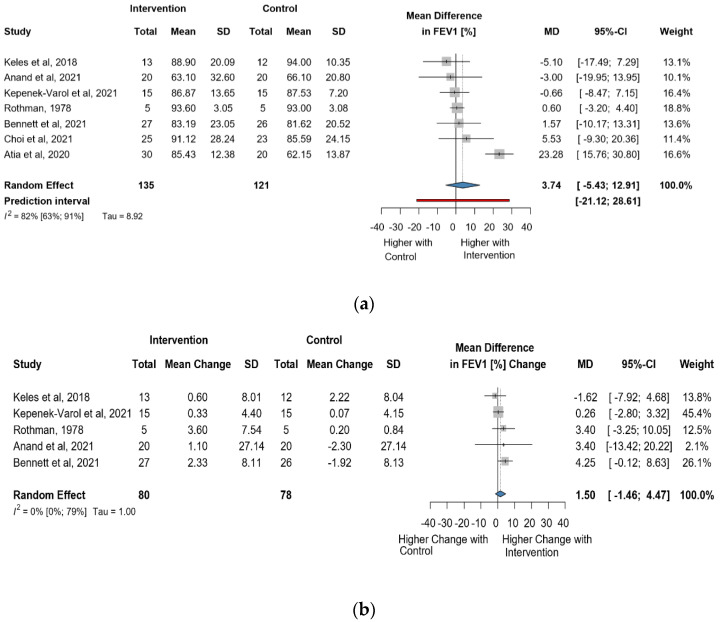
(**a**). Forest plot of FEV1 values in % of cerebral palsy patients after supplementary respiratory therapy plus conventional care versus conventional care. (**b**). Forest plot of direct change in FEV1 values in % of cerebral palsy patients after supplementary respiratory therapy plus conventional care versus conventional care [21,22,23,31,32,33].

Seven studies and 256 observations showed a statistically non-significant change in FEV1(%) (MD = 3.74 [(−)5.43; 12.91] *p* = 0.3565) after the intervention (Figure 5a). Similarly, the evaluation of the direct change after the intervention analyzed in 158 observations indicated a statistically non-significant change (MD = 1.50. [(−)1.45; 4.46] *p* = 0.2317 (Figure 5b). The estimated change in FEV1(%) showed no statistically significant effect MD = (−)4.51 [(−)15.53; 6.49] *p* = 0.3404) (Figure S7).

#### 3.3.3. Change in FEV1/FVC Ratio Due to Intervention

Seven studies and 251 observations showed a statistically non-significant change (MD = 1.44 [(−)3.43; 6.31] *p* = 0.4965) (Figure S1). The supplement contains a comprehensive analysis of the results of the FEV1/FVC ratio (Figures S2 and S3).

#### 3.3.4. Change in PEF Due to Intervention

Nine studies reported data on PEF using liters or percentages as measurement units.

**Figure 6:**
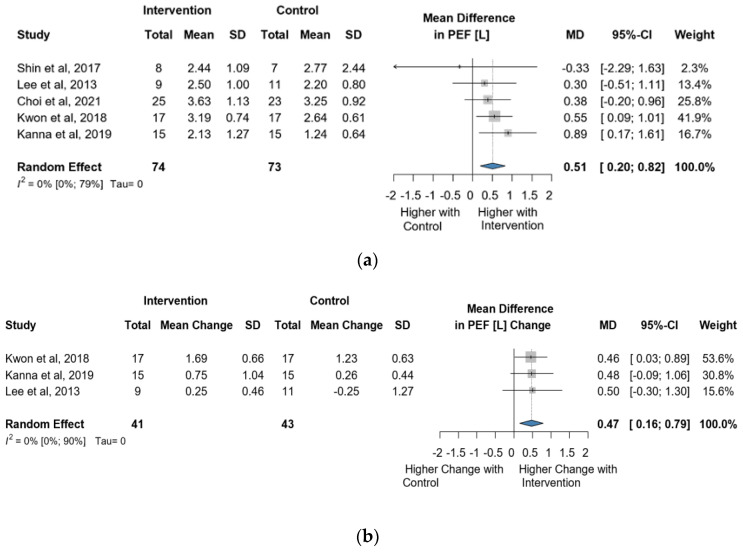
(**a**). Forest plot of PEF values, in liters, of cerebral palsy patients after supplementary respiratory therapy plus conventional care versus conventional care. (**b**). Forest plot of direct change in PEF values, in liters, of cerebral palsy patients after supplementary respiratory therapy plus conventional care versus conventional care [3,4,6,7,8].

Five studies and 147 observations assessed the impact of the intervention on PEF(L) and showed a statistically significant change (MD = 0.51 [0.20; 0.82], *p* = 0.0107) (Figure 6a). Similarly, the direct change analyzed in 84 observations also showed a statistically significant change PEF(L) (MD = 0.47 [0.16; 0.79] *p* = 0.0035) (Figure 6b). The estimated change in PEF(L) showed non statistically significant effect (MD = 2.94 [(−)9.03; 14.92] *p* = 0.4913) (Figure S8).

**Figure 7:**
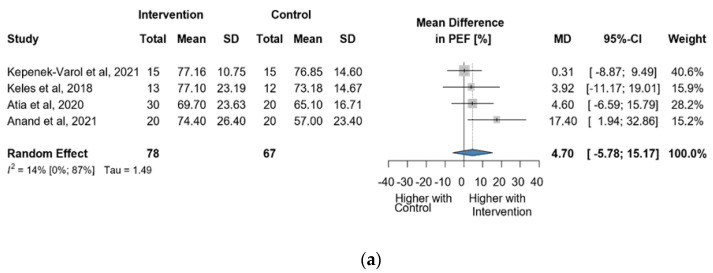

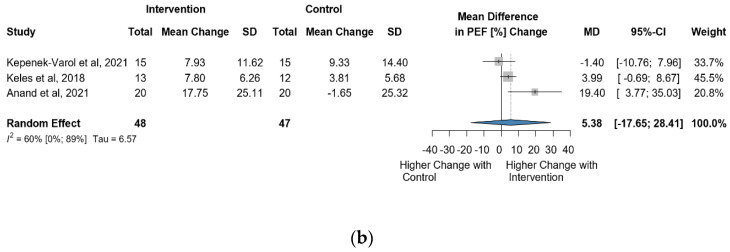
(**a**). Forest plot of direct change in PEF values, in liters, of cerebral palsy patients after supplementary respiratory therapy plus conventional care versus conventional care. (**b**). Forest plot of direct change in PEF values in % of cerebral palsy patients after supplementary respiratory therapy plus conventional care versus conventional care [9,10,11,12].

Four studies and 145 observations showed a statistically non-significant change in PEF (%) MD = 4.70 [(−)5.78; 15.17] *p* = 0.2488) after the intervention (Figure 7a). Similarly, the evaluation of the direct change after the intervention analyzed in 95 observations showed a statistically non-significant change (MD = 5.38 [(−)17.64; 28.41] *p* = 0.4204) (Figure 7b). The estimated change in PEF (%) showed no statistically significant effect MD = 2.94 [(−)9.03; 14.92] *p* = 0.4913) (Figure S9).

#### 3.3.5. Clinical Relevance

We considered the results to be of clinical significance, as the mean changes in FVC, FEV1, and PEF measurements, both in liters and percentages, and FEV1/FVC values, for post-treatment values in the intervention group, direct changes, and estimated changes, exceeded a 5% threshold.

### 3.4. Respiratory Muscle Strength (RMS)

#### 3.4.1. Change in MIP Due to Intervention

**Figure 8:**
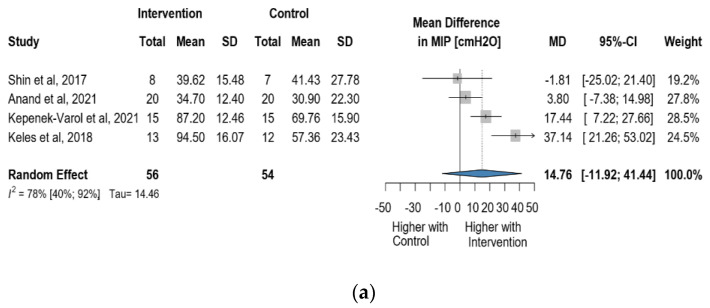

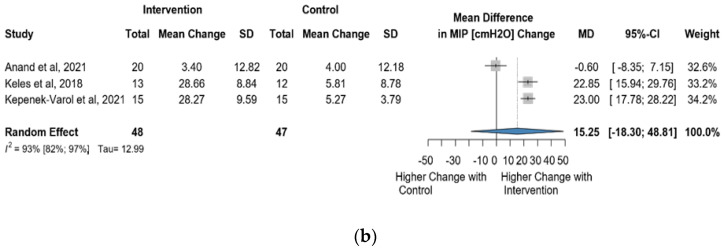
(**a**). Forest plot of MIP values, in cmH_2_O, of cerebral palsy patients after supplementary respiratory therapy plus conventional care versus conventional care. (**b**). Forest plot of direct change in of MIP values, in cmH_2_O, of cerebral palsy patients after supplementary respiratory therapy plus conventional care versus conventional care [29,31,32,33].

Four studies reported data on MIP using cmH_2_O as a measurement unit. Altogether, 110 observations assessed the impact of the intervention on MIP and showed a statistically non-significant change (MD = 14.76 [(−)11.92; 41.44] *p* = 0.1765) (Figure 8a). Similarly, the direct change analyzed in 95 observations also showed a statistically non-significant change MIP MD = 15.25 [(−)18.30; 48.81] *p* = 0.1896) (Figure 8b). The estimated change in MIP showed no statistically significant effect (MD = 15.25 [(−)18.30; 48,81] *p* = 0.1896) (Figure S10).

#### 3.4.2. Change in MEP Due to Intervention

**Figure 9:**
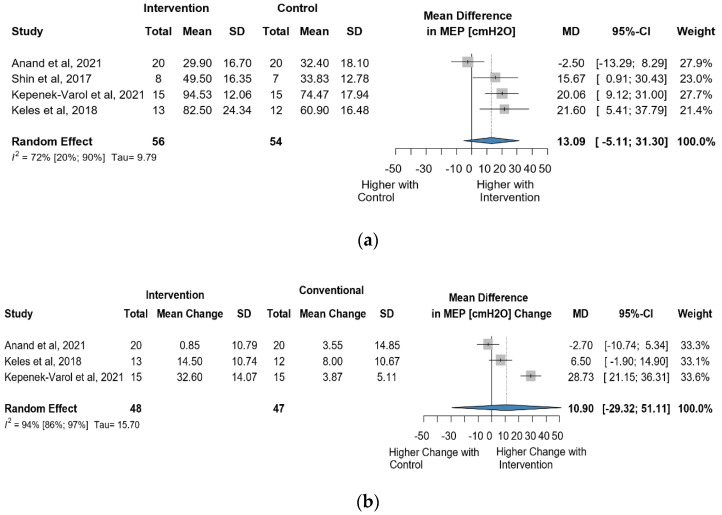
(**a**). Forest plot of MEP values, in cmH_2_O, of cerebral palsy patients after supplementary respiratory therapy plus conventional care versus conventional care. (**b**). Forest plot of direct change in MEP values, in cmH_2_O, of cerebral palsy patients after supplementary respiratory therapy plus conventional care versus conventional care [29,31,32].

Four studies reported data on MEP using cmH_2_O as a measurement unit. A total of 110 observations assessed the impact of the intervention on MEP and showed a statistically non-significant change (MD = 13.09 [(−)5.11; 31.30] *p* = 0.106) (Figure 9a). Similarly, the direct change analyzed in 95 observations also indicated statistically non-significant change MD = 10.89 [(−)29.32; 51.11] *p* = 0.3640) (Figure 9b). The estimated change in MEP showed no statistically significant effect (MD = 4.72 [(−)24.34; 33.79] *p* = 0.6409) (Figure S11).

#### 3.4.3. Clinical Relevance

The post-intervention outcomes, encompassing direct and estimated MEP and MIP changes, are clinically relevant as the mean results of the intervention group consistently exceed the minimum mean difference (MD) threshold of 5%.

### 3.5. Efficacy of Supplementary Respiratory Therapy on Quality of Life (QoL)

Due to insufficient data for a meta-analysis, a literature review was conducted to assess the impact of supplementary respiratory therapy on quality of life.

Keles et al. [32] found significant improvement in cerebral palsy quality of life (CPQOL) in social well-being, acceptance, functioning, and emotional well-being after respiratory therapy. These improvements were influenced by factors such as participation, exercise capacity, and trunk control.

El-Refaey et al. [27] conducted a study in which they compared the PedsQL scores before and after a four-week feedback respiratory training intervention in a study group and a control group. Both groups showed an improvement in PedsQL score, but this improvement was not statistically significant. However, it is important to note the limitations of this study, such as the short duration of the intervention.

Litchke et al. [34] assessed the health-related QoL of wheelchair rugby athletes. This study found significant improvement in certain QoL domains, particularly in bodily pain, and vitality.

### 3.6. Impact of Supplementary Respiratory Therapies on Overall Survival

Our original research design included examining overall survival as an outcome. However, as with previous meta-analyses, we also found no data on this aspect [13,35].

### 3.7. Risk of Bias Assessment

Detailed results of RoB assessments are presented in the Supplementary Material. The quality assessment revealed a severe overall risk of bias for all outcomes and studies. Bias due to the randomization process was the main source of potential bias (Figure S18).

### 3.8. Quality of Evidence

Given the limited sample size of the studies analyzed, publication bias was assessed by visual inspection of funnel plots, which provide no evidence for the presence of publication bias (Figures S14–S17). The GRADE (Grading of Recommendations Assessment, Development, and Evaluation) system scores the quality of evidence as high to moderate for pulmonary function and low for respiratory muscle strength (Table S3).

## 4. Discussion

This study aimed to investigate the effects of incorporating supplementary respiratory therapy into standard care versus standard care alone, and therefore we analyzed pulmonary function, respiratory muscle strength, and quality of life in patients with CP.

Our analysis revealed a statistically significant difference between the intervention and control groups concerning FEV1(L) and PEF(L) measurements. Although we did not find any statistically significant changes in FVC, FEV1 (%), FEV1/FVC ratio, PEF (%), MIP, and MEP, we consider the increase in pulmonary function tests and respiratory muscle strength after the intervention to be clinically significant.

Given the absence of guidance on the CP-specific minimal clinically important difference (MCID) guidance and drawing on MCID values in other lung diseases, our observations indicate an increase of approximately 5% as the minimal clinically important difference [35,36,37]. This indicates clinical significance, where changes—though not always statistically significant—hold meaning, and an increase of at least 5% after intervention is considered clinically relevant.

The studies included in our meta-analysis had to be further disaggregated according to reported data in liters, whereas others reported data in percentages. A previous study reported statistically significant differences between the experimental and control groups in PEF and FEV1 after respiratory therapy; however, although that study combined percentage (%) and liter (L)-based measures in the analysis, we analyzed them separately in our study [38].

Our findings align with those of a previous meta-analysis, which showed that respiratory muscle training (RMT) interventions effectively improved respiratory function in adults with post-stroke conditions, having similar etiology and symptoms to cerebral palsy (CP) [39].

The studies used six types of supplementary respiratory therapy, including manual diaphragmatic stretching technique [21], incentive spirometer [14,33], feedback respiratory training [29,30], inspiratory muscle training (IMT) [24,25,26], breathing exercise series [27,28], and resistance exercise [34,40].

The most effective dose of supplementary respiratory therapy for pediatric patients with CP is currently unknown, reflecting the wide variation in the duration and length of the studies included in this analysis [12]. Duration varied considerably, ranging from four weeks to 12 weeks. The crucial issue of compliance arises, especially for children with CP, who generally require additional motivation and encouragement to actively participate in all activities [40,41].

In terms of compliance, only Anand et al. highlighted that IMT is monotonous and may not be suitable for children, as they do not find it enjoyable, Rutka et al. considered IMT to be promising and effective, especially in developing the RMS [33,41]. A study by McCoy et al. demonstrates the effectiveness of various therapies for children with CP [42]. Play and activity-based approaches are particularly beneficial for surpassing expected progress [42,43]. This suggests that tailoring supplementary therapy to individualized, age-appropriate, and playful concepts, aligned with the interests of children and encouraging active involvement, may enhance its effectiveness.

Based on available data, interventions involving individuals with GMFCS levels II–IV for more than six weeks generally appear to result in greater improvements in favor of the intervention group, particularly in pulmonary function tests consistent with previous observations that children classified at GMFCS level I–II might have already attained their maximum respiratory function capacity, resulting in no relevant improvements after supplementary respiratory therapy [22,33,41,44].

Performing spirometry in patients with CP can be challenging due to disease severity, especially in conditions such as hemiplegia or weakness of the orofacial muscles [5,45]. Consequently, subjects included CP patients who could undergo spirometry, leading to the exclusion of several potential individuals with CP who may not have been able to participate in the measurement. Furthermore, it is known that the PFTs can vary depending on factors such as height, weight, sex, and even body position, such as standing or sitting, and supine position is recommended in addition to sitting [46].

The body position during PFTs was unknown in four of the included studies [24,25,31,32], whereas in the remaining others, measurements were consistently taken in a sitting and corrected body position.

PEF is considered an easy-to-perform measure of pulmonary function and it is less affected by factors such as lung volume, body position, or co-operation, ensuring reliability and objectivity [47,48]. As for the unique characteristics of this value, it is worth considering its utilization in educational, rehabilitation, or home settings for individuals with CP. Teaching individuals with CP to use this value would help self-monitoring and facilitate the establishment of appropriate therapeutic interventions.

Evidence may support the existence of a positive association between enhanced respiratory status and improved QoL in pediatric patients with asthma and lung disease, which is potentially relevant in CP [45]. A meta-analysis of this topic could not be conducted at present due to lack of data.

### 4.1. Strengths

Only RCTs were included, and a rigorous methodology was followed. We took great care to avoid any duplicate publications [23,28]. We tried to overcome the difficulties arising from the different ways in which research was presented by fitting several models. Our analysis is detailed and complex because, in addition to analyzing values measured after the intervention, it also includes calculations for the immediate change and estimated change to give a more accurate picture of the magnitude of the impact of the intervention.

Our meta-analysis demonstrates a significant effort to determine the clinical significance of the observed increases in each value. The 5% increment, calculated as the minimal clinically important difference, provides valuable insights in addition to statistical significance, making our findings more meaningful for healthcare professionals and patients.

### 4.2. Limitations

The results of this systematic review should be interpreted cautiously in the context of a relatively small number of participants with heterogeneous interventions which reduces the certainty of the findings, although small sample sizes are common in studies of this nature.

The RCTs analyzed were conducted globally and included patients with varying levels of gross motor function classification system (GMFCS) I–IV and different diagnoses of cerebral palsy, resulting in a heterogeneous and incomplete patient population (GMFCS V not represented), aged 6–18 years. Furthermore, the generalizability of the results may be affected by the fact that certain studies failed to include the GMFCS levels [24,25,32] which can be a significant determinant of respiratory status [49,50].

The assessment of the quality of evidence revealed several methodological limitations, including the impossibility of blinding and the absence of an obvious randomization process. Publication bias did not lead to downgrading of studies due to limited trials, as direct evidence was insufficient for any specific intervention.

We need to take into consideration this aspect for proper interpretation of the results. Collectively, these limitations undermine confidence in the results, resulting in moderate pulmonary function tests (PFTs) and low-quality evidence for respiratory muscle strength (RMS) (Table S3).

### 4.3. Implications for Practice

The benefits of promptly implementing scientific findings have already been demonstrated [51,52]. Our findings suggest that healthcare professionals should manage pediatric patients with CP by incorporating individualized respiratory therapies into the rehabilitation plan of children. Tailoring complementary respiratory therapy to individual needs provides comprehensive and sustainable care for pediatric patients with CP. Tailoring therapy based on factors such as diagnosis, age, severity, motivation, and psychological aspects meets unique requirements, promotes engagement, and provides a holistic approach.

Our research aligns with the recommendations of previous studies [11,12,41], calling for the inclusion of the most vulnerable group, GMFCS V, in similar studies conducted in the future [49].

### 4.4. Implications for Future Research

To ensure a more precise and meaningful interpretation of treatment outcomes, future research should prioritize defining the MCID for each GMFCS level and diagnosis.

To provide a more comprehensive understanding of pulmonary function and facilitate thorough analyses and comparisons between interventions, future studies should incorporate both liter-based and percentage-based values.

To better understand the effectiveness of interventions and to allow for more accurate interpretations and comparisons of study results, future studies should prioritize comprehensive reporting of protocols used in the comparison group.

We suggest conducting longitudinal cohort studies to comprehensively assess the effectiveness of respiratory interventions, their impact on mortality, morbidity, and quality of life, and the long-term consequences of discontinuing therapy.

## 5. Conclusions

This meta-analysis provides the most up-to-date evidence that in the pediatric population with cerebral palsy GMFCS level I-IV, the addition of supplementary respiratory therapies to standard treatment has a clinically significant impact on improving pulmonary function and respiratory muscle strength.

This promising approach has the potential to improve quality of life and overall survival outcomes.

Based on our results and the previous analysis, these findings are synergistically supportive and may contribute to developing a new guideline on the incorporation of respiratory therapies into standard care for individuals with cerebral palsy.

## Supplementary Materials

The following supporting information can be downloaded at: https://www.mdpi.com/article/10.3390/jcm13030888/s1. The following supporting information can be found in Supplementary Material: Table of contents: Detailed description of synthesis methods. Figure S1. Forest plot of change of FEV1/FVC% of cerebral palsy patients after supplementary respiratory therapy plus conventional care versus conventional care alone. Figure S2. Forest plot of direct change of FEV1/FVC% of cerebral palsy patients after supplementary respiratory therapy plus conventional care versus conventional care alone. Figure S3. Forest plot of estimated change of FEV1/FVC% cerebral palsy patients after supplementary respiratory therapy plus conventional care versus conventional care alone. Figure S4. Forest plot of estimated change of FVC, in liters, of cerebral palsy patients after supplementary respiratory therapy plus conventional care versus conventional care. Figure S5. Forest plot of estimated change of FVC in % of cerebral palsy patients after supplementary respiratory therapy plus conventional care versus conventional care alone. Figure S6. Forest plot of estimated change of FEV1, in liters, of cerebral palsy patients after supplementary respiratory therapy plus conventional care versus conventional care alone. Figure S7. Forest plot of estimated change of FEV1 in % of cerebral palsy patients after supplementary respiratory therapy plus conventional care versus conventional care alone. Figure S8. Forest plot of estimated change of PEF, in liters, of cerebral palsy patients after supplementary respiratory therapy plus conventional care versus conventional care alone. Figure S9. Forest plot of estimated change of PEF in % of cerebral palsy patients after supplementary respiratory therapy plus conventional care versus conventional care alone. Figure S10. Forest plot of estimated change of MIP values in cmH_2_O of cerebral palsy patients after supplementary respiratory therapy plus conventional care versus conventional care alone. Figure S11. Forest plot of estimated change of MEP values in cmH_2_O of cerebral palsy patients after supplementary respiratory therapy plus conventional care versus conventional care alone. Figure S12. Multivariate analysis of FVC % and FEV1% values of cerebral palsy patients after supplementary respiratory therapy plus conventional care versus conventional care alone. Figure S13. Multivariate analysis of MIP and MEP values of cerebral palsy patients after supplementary respiratory therapy plus conventional care versus conventional care alone. Figure S14. Funnel plot of studies with FEV1/FVC% outcome after supplementary respiratory therapy plus conventional care versus conventional care alone. Figure S15. Funnel plot of studies with FVC (a). in liters and (b). FVC in percentage outcome after supplementary respiratory therapy plus conventional care versus conventional care alone. Figure S16. Funnel plot of studies with FEV1 (a). in liters and (b). FEV1 in percentage outcome after supplementary respiratory therapy plus conventional care versus conventional care alone. Figure S17. Funnel plot of studies with PEF (a). in liters and (b). PEF in percentage outcome after supplementary respiratory therapy plus conventional care versus conventional care alone. Figure S18. Risk of bias assessment (Rob2 tool) Outcome: pulmonary functions and respiratory strength. Figure S19. Leave-one-out analyses, sorted by effect size plots of studies with FVC outcomes. Figure S20. Leave-one-out analyses, sorted by effect size plots of studies with FEV1 outcomes. Figure S21. Leave-one-out analyses, sorted by effect size plots of studies with PEF outcomes. Figure S22. Leave-one-out analyses, sorted by effect size plots of studies with RMS outcomes. Table S1. Baseline characteristics of the included trials. Table S2. Search key. Table S3. Summary of findings: Grading of Recommendations, Assessment, Development and Evaluations (GRADE) framework. Table S4. PRISMA 2020 Checklist. References [52,53,54,55,56] are cited within Supplementary Materials.
