# Supplementary material for: Supplementary Respiratory Therapy Improves Pulmonary Function in Pediatric Patients with Cerebral Palsy: A Systematic Review and Meta-Analysis
Source: J Clin Med. 2024 Feb 2;13(3):888. doi: 10.3390/jcm13030888 (PMC10856351; doi:10.3390/jcm13030888)
Supplement: Supplementary file 1 [file jcm-13-00888-s001.zip › jcm-2809276-supplementary.pdf]

Supplementary Materials

Title: Supplementary Respiratory Therapy Improves Pulmonary Function in Paediatric Patients with Cerebral Palsy: A Systematic Review and Meta-analysis

Erika Kolumbán[1,2], Márton Szabados[1,7], Márk Hernádfői[1,4], Uyen Nguyen Do To[2], Rita Nagy[1,3,5], Ádám Zolcsák[1,8], Katalin Eszter Müller[3,5,9], Zoltán Sipos[5,10], Dániel Sándor Veres[1,8], Anett Szőllősi[4], Péter Hegyi[1,5,6], Miklós Garami[1,7], Ibolya Túri[1,2]

Affiliations:

1. Centre for Translational Medicine, Semmelweis University, Budapest, Hungary
2. András Pető Faculty, Semmelweis University, Budapest, Hungary
3. Heim Pál National Pediatric Institute, Budapest, Hungary
4. Bethesda Children's Hospital, Budapest, Hungary
5. Institute for Translational Medicine, Medical School, University of Pécs, Pécs, Hungary
6. Institute of Pancreatic Diseases, Semmelweis University, Budapest, Hungary.
7. Pediatric Center, Semmelweis University, Budapest, Hungary
8. Department of Biophysics and Radiation Biology, Semmelweis University, Budapest, Hungary
9. Department of Family Care Methodology, Faculty of Health Sciences, Semmelweis University, Budapest, Hungary
10. Institute of Bioanalysis, Medical school, University of Pécs, Pécs, Hungary

Corresponding author

Erika Kolumbán, MD

Postal address: H-1125 Budapest, Kútvölgyi str. 6., Hungary

Tel.: +36 1 224-1500

E-mail address: kolumban.erika@semmelweis.hu

Table of contents:

Detailed description of synthesis methods

Figure S1. Forest plot of change of FEV1/FVC% of cerebral palsy patients after supplementary respiratory therapy plus conventional care versus conventional care alone

Figure S2. Forest plot of direct change of FEV1/FVC% of cerebral palsy patients after supplementary respiratory therapy plus conventional care versus conventional care alone

Figure S3. Forest plot of estimated change of FEV1/FVC% cerebral palsy patients after supplementary respiratory therapy plus conventional care versus conventional care alone

Figure S4. Forest plot of estimated change of FVC in litre of cerebral palsy patients after supplementary respiratory therapy plus conventional care versus conventional care

Figure S5. Forest plot of estimated change of FVC in % of cerebral palsy patients after supplementary respiratory therapy plus conventional care versus conventional care alone

Figure S6. Forest plot of estimated change of FEV1 in litre of cerebral palsy patients after supplementary respiratory therapy plus conventional care versus conventional care alone

Figure S7. Forest plot of estimated change of FEV1 in % of cerebral palsy patients after supplementary respiratory therapy plus conventional care versus conventional care alone

Figure S8. Forest plot of estimated change of PEF in litre of cerebral palsy patients after supplementary respiratory therapy plus conventional care versus conventional care alone

Figure S9. Forest plot of estimated change of PEF in % of cerebral palsy patients after supplementary respiratory therapy plus conventional care versus conventional care alone

Figure S10. Forest plot of estimated change of MIP values in cmH2O of cerebral palsy patients after supplementary respiratory therapy plus conventional care versus conventional care alone

Figure S11. Forest plot of estimated change of MEP values in cmH2O of cerebral palsy patients after supplementary respiratory therapy plus conventional care versus conventional care alone

Figure S12. Multivariate analysis of FVC % and FEV1 % values of cerebral palsy patients after supplementary respiratory therapy plus conventional care versus conventional care alone

Figure S13. Multivariate analysis of MIP and MEP values of cerebral palsy patients after supplementary respiratory therapy plus conventional care versus conventional care alone

Figure S14. Funnel plot of studies with FEV1/FVC% outcome after supplementary respiratory therapy plus conventional care versus conventional care alone

Figure S15. Funnel plot of studies with FVC a.) in litre and b.) FVC in percentage outcome after supplementary respiratory therapy plus conventional care versus conventional care alone

Figure S16. Funnel plot of studies with FEV1 a.) in litre and b.) FEV1 in percentage outcome after supplementary respiratory therapy plus conventional care versus conventional care alone

Figure S17. Funnel plot of studies with PEF a.) in litre and b.) PEF in percentage outcome after supplementary respiratory therapy plus conventional care versus conventional care alone

Figure S18. Risk of bias assessment (Rob2 tool) Outcome: pulmonary functions and respiratory strength

Figure S19. Leave-one-out analyses, sorted by effect size plots of studies with FVC outcomes.(a) in percentage (b) in liter

Figure S20 Leave-one-out analyses, sorted by effect size plots of studies with FEV1 outcomes.(a) in percentage (b) in liter

Figure S21. Leave-one-out analyses, sorted by effect size plots of studies with PEF outcomes.(a) in percentage (b) in liter.

Figure S22. Leave-one-out analyses, sorted by effect size plots of studies with RMS outcomes.(a) in MEP (b) in MIP

Table S1. Baseline characteristics of the included trials

Table S2. Search key

Table S3. Summary of findings: Grading of Recommendations, Assessment, Development and Evaluations (GRADE) framework

Table S4. PRISMA 2020 Checklist

Detailed description of synthesis methods

The meta-analysed articles contained mean and SD values for before and after the intervention or change during intervention. In some articles the change for the intervention was not given with mean and SD, but a paired t-test t-value, or a CI of the change mean based on a t-test was given, therefore we could calculate the mean and SD. Inverse variance weighting method was used to calculate the pooled MD. We used a Hartung-Knapp adjustment,[1,2] CIs. This adjustment was applied only if it is more conservative than the classical one (as recommended by Jackson et al.[3] as hybrid method 2). To estimate the heterogeneity variance measure (τ^2), the restricted maximum-likelihood estimator was used with the Q profile method for confidence interval.[4,5] Prediction interval calculations were based on a Hartung-Knapp adjustment too. On the forest plots, t-distribution-based method used for CI of MD calculation of individual studies.

As in one hand, several studies reported the observed values before (baseline) and after the treatment, but the change was not reported, and on the other hand, several studies reported the change without reporting the baseline and after treatment values, therefore the syntheses of all together is not possible without additional assumptions. Regarding to this situation, we performed different analyses to estimate the effect and its significance.

1.) Where the after or change values are "directly" reported, we used these values separately for pooling. We reported the change values as after minus before intervention values. As we have only RCT studies, we could assume that the theoretical before value of mean difference between intervention and control group is 0. In this case no additional assumption needed for the change calculation.

2.) To pool together all the study results, we estimated the mean and SD of change from baseline to after treatment where it is not reported. For this, we should estimate the the correlation coefficient (R) between baseline and after values. The estimation of R was based on the extracted or calculated SD of change if the SD for baseline and after value was also given in the study - from where the correlation coefficient is calculable. The mean of the calculated R values was used as estimand of R for estimating change values. After estimating change mean and SD where it is not given (imputing the R), we pool together the directly given and estimated change values. If no study contained information on R, we used the value 0.5. Additionally, we made these analyses with assuming different R values – as influental analyses - to see how it influence the pooled effect.

3.) We used a random-effects multivariate model with a restricted maximum likelihood method on before and after values. For within-studies correlation (sampling error covariance matrix), the previously mentioned estimated R was used in a block-diagonal structure. A compound symmetry structure was assumed for dependency between true effects. We performed the multivariate model and the result was modified with cluster-robust test and confidence interval estimation adjustment using clubsandwich method with small-sample and Satterthwaite approximation. As sensitivity analysis, we run the model with different correlation coefficient values to see how it influence the pooled effect. We did not report these results as it provided not substantially different results as in model 2.).

A random-effects multivariate model was built on FVC - FEV (expressed in percentages) and MIP-MEP outcomes. For within studies correlation (sampling error covariance matrix), R=0.5 was used in a block-diagonal structure. A compound symmetry structure was assumed for dependency between true effects. We performed the multivariate model and the result was modified with cluster-robust test and confidence interval estimation adjustment using clubsandwich method with small-sample and Satterthwaite approximation. As a sensitivity analysis, we run the model with different correlation coefficient values to see how it influences the pooled effect.
In all situations, where we used R values, the sensitivity analyses revealed that the value of R did not changed the results in a relevant amount.

Plots with leave-one-out analysis values:

In leave-on-out analysis we calculate the parameters of interest excluding the studies one-by-one.
The assessed parameters are:

- Θ: effect size: *the pooled effect* size without the given study.
- 95% CI: *the 95% confidence interval* of the pooled effect size without the given study.
- I^2: *the Higgins&Thomson I^2 heterogeneity* value without the given study.
- Stand. residual: *the studentized residuals*. It shows the deleted residual divided by its estimated standard deviation.
- Dffits: *the difference in fits*. It quantifies the number of standard deviations that the fitted value changes without the given study. (Typical threshold is $3 * \sqrt{\frac{p}{k-p}}$, where p is the number of model coefficients and k the number of cases)
- Cook's Distance: *Cook's distance*. It depends on both the residual and leverage of the omitted study. (Typical threshold value is 2)
- Covariance Ratio: *the covariance ratio*. It shows the change in the determinant of the covariance matrix of the effect size. (Typical threshold value is 1)
- Tau-squared: *the square of tau value* (random effects variance, between study heterogeneity). Tau-squared value without the given study.
- Q: *test statistics of tau-squared*. Q value without the given study.
- Hat value: *the value of the hat matrix* without the given study. (Typical threshold is $3 * \frac{p}{k}$)
- weight: *study weight* in the analysis.

Figure S1. Forest plot of change of FEV1/FVC% of cerebral palsy patients after supplementary respiratory therapy plus conventional care versus conventional care alone [31,22,23,21,24,20,26].

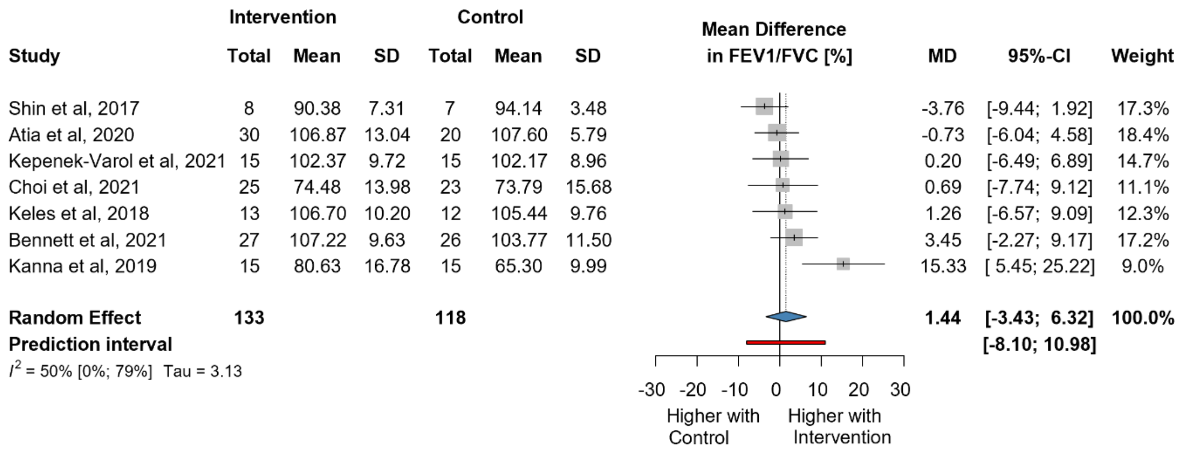

Figure S2. Forest plot of direct change of FEV1/FVC% of cerebral palsy patients after supplementary respiratory therapy plus conventional care versus conventional care alone [23,20,24,26]

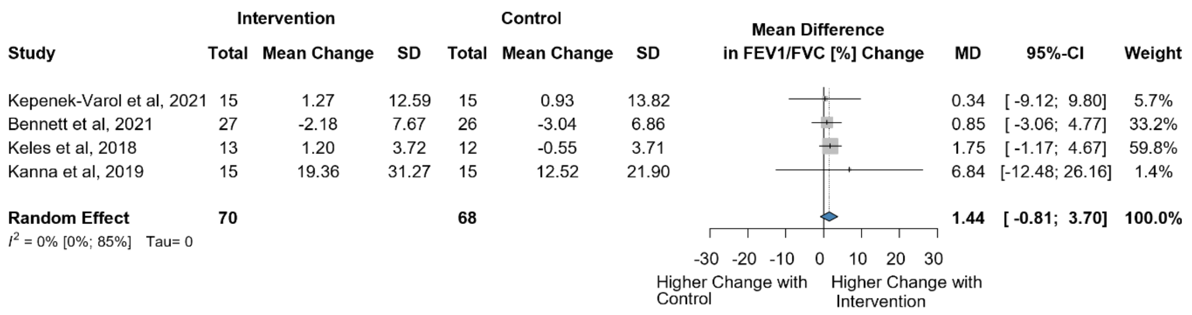

Figure S3. Forest plot of estimated change of FEV1/FVC% cerebral palsy patients after supplementary respiratory therapy plus conventional care versus conventional care alone [23,21,31,20,22,24,26]

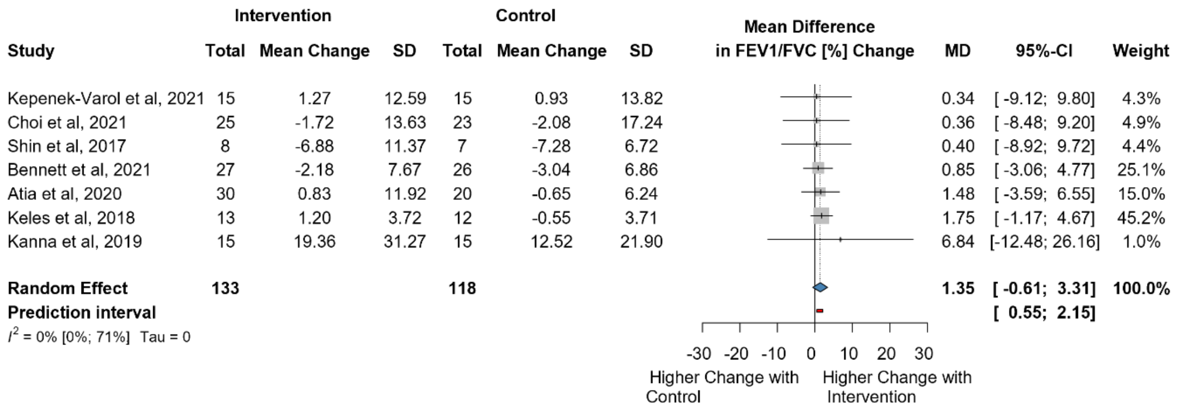

Figure S4. Forest plot of estimated change of FVC in litre of cerebral palsy patients after supplementary respiratory therapy plus conventional care versus conventional care alone [21,31,20, 32,28,26]

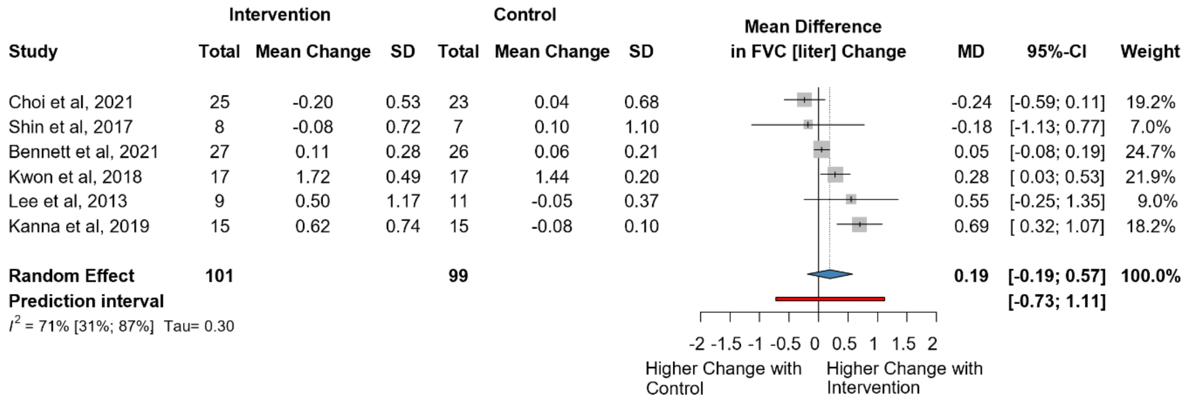

Figure S5. Forest plot of estimated change of FVC in % of cerebral palsy patients after supplementary respiratory therapy plus conventional care versus conventional care alone [22,21,25,23,24,20]

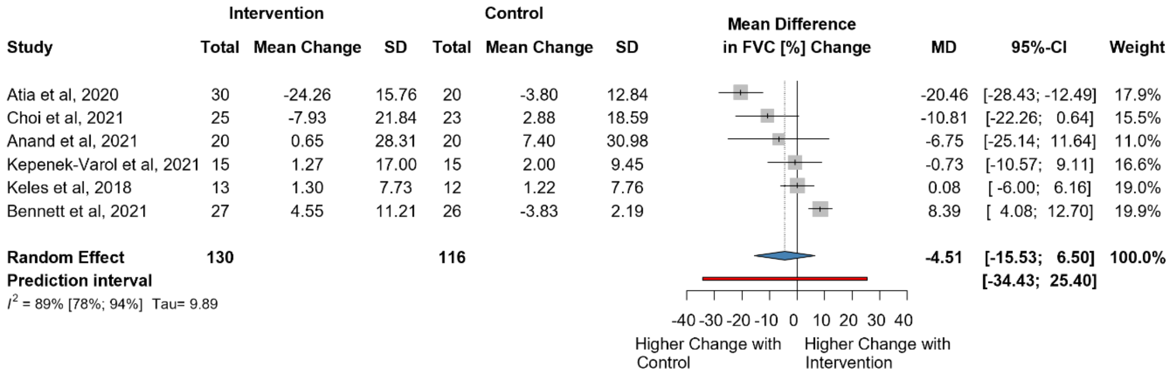

Figure S6. Forest plot of estimated change of FEV1 in litre of cerebral palsy patients after supplementary respiratory therapy plus conventional care versus conventional care alone [21,31,20,26,28,32]

Study	Intervention Total	Intervention Mean Change	Intervention SD	Control Total	Control Mean Change	Control SD	MD	95%-CI	Weight
Choi et al, 2021	25	-0.17	0.42	23	0.00	0.46	-0.17	[-0.42; 0.08]	24.0%
Shin et al, 2017	8	-0.17	0.60	7	-0.06	0.97	-0.11	[-0.94; 0.72]	5.0%
Bennett et al, 2021	27	0.05	0.20	26	-0.00	0.19	0.06	[-0.05; 0.16]	34.9%
Kanna et al, 2019	15	0.54	0.58	15	0.27	0.46	0.27	[-0.10; 0.65]	16.4%
Lee et al, 2013	9	0.40	0.91	11	0.10	0.00	0.30	[-0.29; 0.89]	8.7%
Kwon et al, 2018	17	1.69	0.62	17	1.15	0.88	0.54	[0.03; 1.05]	10.9%
**Random Effect**	**101**			**99**			**0.10**	**[-0.15; 0.36]**	**100.0%**
**Prediction interval**								**[-0.43; 0.64]**	

I^2 = 42% [0%; 77%] Tau= 0.16

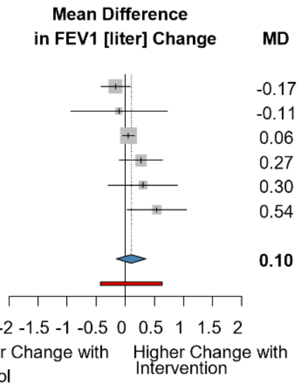

Figure S7. Forest plot of estimated change of FEV1 in % of cerebral palsy patients after supplementary respiratory therapy plus conventional care versus conventional care alone [22,21,24,23,27,25,20]

Study	Intervention Total	Intervention Mean Change	Intervention SD	Control Total	Control Mean Change	Control SD	MD	95%-CI	Weight
Atia et al, 2020	30	-25.26	13.26	20	-4.00	10.56	-21.26	[-27.89; -14.63]	15.2%
Choi et al, 2021	25	-9.21	21.02	23	-1.17	18.04	-8.04	[-19.10; 3.02]	12.2%
Keles et al, 2018	13	0.60	8.01	12	2.22	8.04	-1.62	[-7.92; 4.68]	15.4%
Kepenek-Varol et al, 2021	15	0.33	4.40	15	0.07	4.15	0.26	[-2.80; 3.32]	16.9%
Rothman, 1978	5	3.60	7.54	5	0.20	0.84	3.40	[-3.25; 10.05]	15.1%
Anand et al, 2021	20	1.10	27.14	20	-2.30	27.14	3.40	[-13.42; 20.22]	8.8%
Bennett et al, 2021	27	2.33	8.11	26	-1.92	8.13	4.25	[-0.12; 8.63]	16.4%
**Random Effect**	**135**			**121**			**-2.90**	**[-11.50; 5.70]**	**100.0%**
**Prediction interval**								**[-26.81; 21.01]**	

I^2 = 87% [75%; 93%] Tau= 8.61

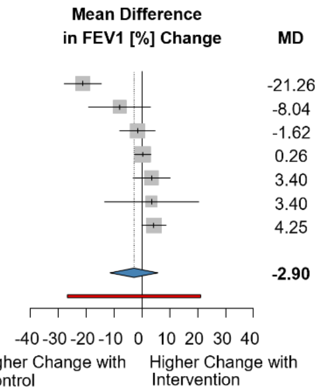

Figure S8. Forest plot of estimated change of PEF in litre of cerebral palsy patients after supplementary respiratory therapy plus conventional care versus conventional care alone [21,31,32,26,28]

Study	Intervention Total	Intervention Mean Change	Intervention SD	Control Total	Control Mean Change	Control SD	MD	95%-CI	Weight
Choi et al, 2021	25	-0.43	1.00	23	-0.15	0.92	-0.28	[-0.82; 0.26]	25.3%
Shin et al, 2017	8	-0.46	1.01	7	-0.28	2.16	-0.18	[-1.93; 1.57]	4.2%
Kwon et al, 2018	17	1.69	0.66	17	1.23	0.63	0.46	[0.03; 0.89]	31.3%
Kanna et al, 2019	15	0.75	1.04	15	0.26	0.44	0.48	[-0.09; 1.06]	23.8%
Lee et al, 2013	9	0.25	0.46	11	-0.25	1.27	0.50	[-0.30; 1.30]	15.4%
**Random Effect**	**74**			**73**			**0.26**	**[-0.21; 0.73]**	**100.0%**

I^2 = 31% [0%; 74%] Tau = 0.26

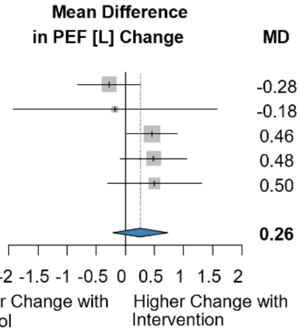

Figure S9. Forest plot of estimated change of PEF in % of cerebral palsy patients after supplementary respiratory therapy plus conventional care versus conventional care alone [23,24,25]

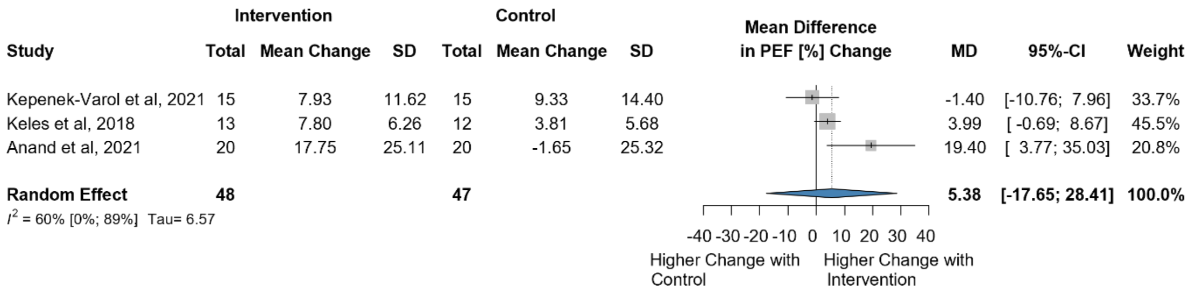

Figure S10. Forest plot of estimated change of MIP values in cmH2O of cerebral palsy patients after supplementary respiratory therapy plus conventional care versus conventional care alone [25,24,23,31]

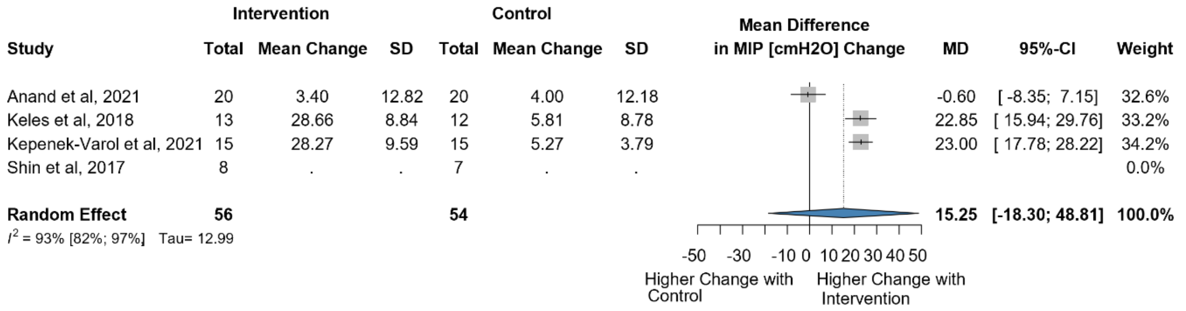

Figure S11. Forest plot of estimated change of MEP values in cmH2O of cerebral palsy patients after supplementary respiratory therapy plus conventional care versus conventional care alone [31,25,24,23]

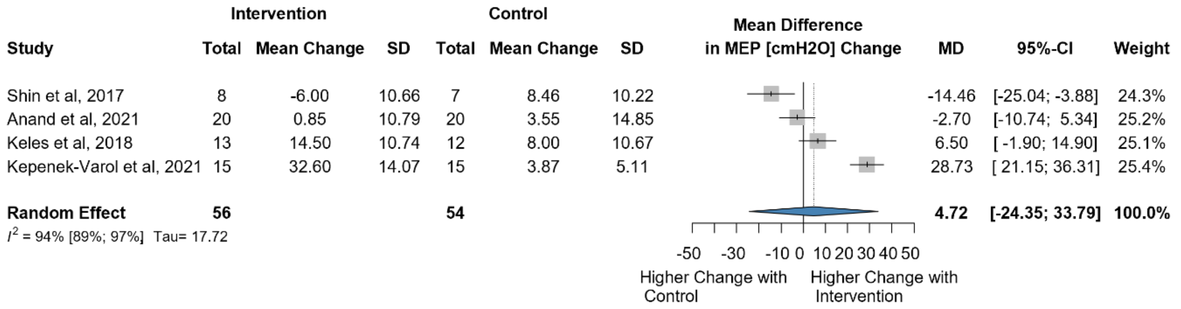

Figure S12. Multivariate analysis of FVC % and FEV1 % values of cerebral palsy patients after supplementary respiratory therapy plus conventional care versus conventional care

alone [24,25,23,27,20,21,22]

Study	Intervention Total	Intervention Mean	Intervention SD	Control Total	Control Mean	Control SD	MD	95%-CI	Weight
**FEV1**									
Keles et al, 2018	13	88.90	20.09	12	94.00	10.35	-5.10	[-17.49; 7.29]	13.1%
Anand et al, 2021	20	63.10	32.60	20	66.10	20.80	-3.00	[-19.95; 13.95]	10.2%
Kepenek-Varol et al, 2021	15	86.87	13.65	15	87.53	7.20	-0.66	[-8.47; 7.15]	16.4%
Rothman, 1978	5	93.60	3.05	5	93.00	3.08	0.60	[-3.20; 4.40]	18.7%
Bennett et al, 2021	27	83.19	23.05	26	81.62	20.52	1.57	[-10.17; 13.31]	13.6%
Choi et al, 2021	25	91.12	28.24	23	85.59	24.15	5.53	[-9.30; 20.36]	11.5%
Atia et al, 2020	30	85.43	12.38	20	62.15	13.87	23.28	[15.76; 30.80]	16.6%
**Random Effect**	**135**			**121**			**3.35**	**[-6.25; 12.95]**	**100.0%**
**Prediction interval**								**[-21.12; 28.61]**	
**FVC1**									
Anand et al, 2021	20	60.80	28.20	20	70.60	20.90	-9.80	[-25.18; 5.58]	13.4%
Keles et al, 2018	13	81.60	17.84	12	86.00	12.10	-4.40	[-16.27; 7.47]	16.2%
Bennett et al, 2021	27	86.37	26.22	26	86.58	19.09	-0.21	[-12.52; 12.10]	15.8%
Kepenek-Varol et al, 2021	15	83.33	12.76	15	82.91	10.08	0.42	[-7.81; 8.65]	19.3%
Choi et al, 2021	25	92.18	24.98	23	83.31	18.44	8.87	[-3.49; 21.23]	15.8%
Atia et al, 2020	30	80.43	14.27	20	58.35	13.93	22.08	[14.12; 30.04]	19.6%
Rothman, 1978	.	.	.	.	.	.			0.0%
**Random Effect**	**130**			**116**			**3.08**	**[-6.54; 12.70]**	**100.0%**
**Prediction interval**								**[-27.46; 34.65]**	

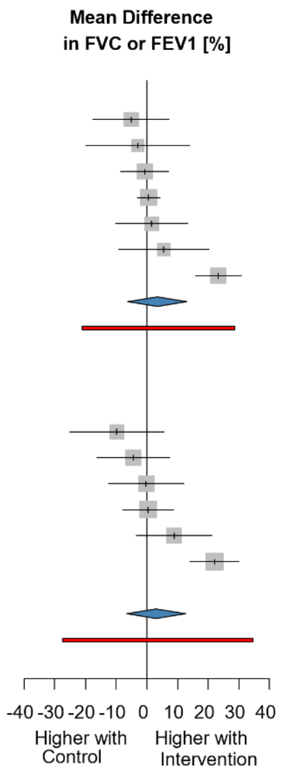

Figure S13. Multivariate analysis of MIP and MEP values of cerebral palsy patients after supplementary respiratory therapy plus conventional care versus conventional care alone [25,31,23,24]

Study	Intervention Total	Intervention Mean	Intervention SD	Control Total	Control Mean	Control SD	MD	95%-CI	Weight
**MEP**									
Anand et al, 2021	20	29.90	16.70	20	32.40	18.10	-2.50	[-13.29; 8.29]	27.5%
Shin et al, 2017	8	49.50	16.35	7	33.83	12.78	15.67	[0.91; 30.43]	23.3%
Kepenek-Varol et al, 2021	15	94.53	12.06	15	74.47	17.94	20.06	[9.12; 31.00]	27.4%
Keles et al, 2018	13	82.50	24.34	12	60.90	16.48	21.60	[5.41; 37.79]	21.8%
**Random Effect**	**56**			**54**			**13.75**	**[-5.59; 33.09]**	**100.0%**
**MIP**									
Shin et al, 2017	8	39.62	15.48	7	41.43	27.78	-1.81	[-25.02; 21.40]	16.8%
Anand et al, 2021	20	34.70	12.40	20	30.90	22.30	3.80	[-7.38; 14.98]	29.1%
Kepenek-Varol et al, 2021	15	87.20	12.46	15	69.76	15.90	17.44	[7.22; 27.66]	30.2%
Keles et al, 2018	13	94.50	16.07	12	57.36	23.43	37.14	[21.26; 53.02]	23.8%
**Random Effect**	**56**			**54**			**15.48**	**[-9.08; 40.05]**	**100.0%**

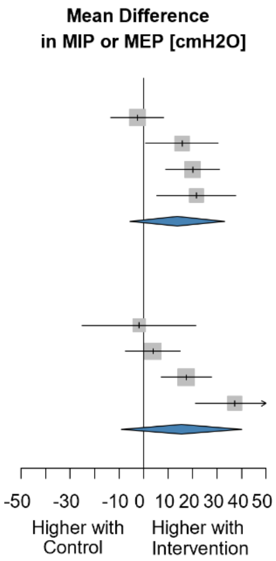

Figure S14. Funnel plot of studies with FEV1/FVC% outcome after supplementary respiratory therapy plus conventional care versus conventional care alone [22,31,20,23,24,21,26]

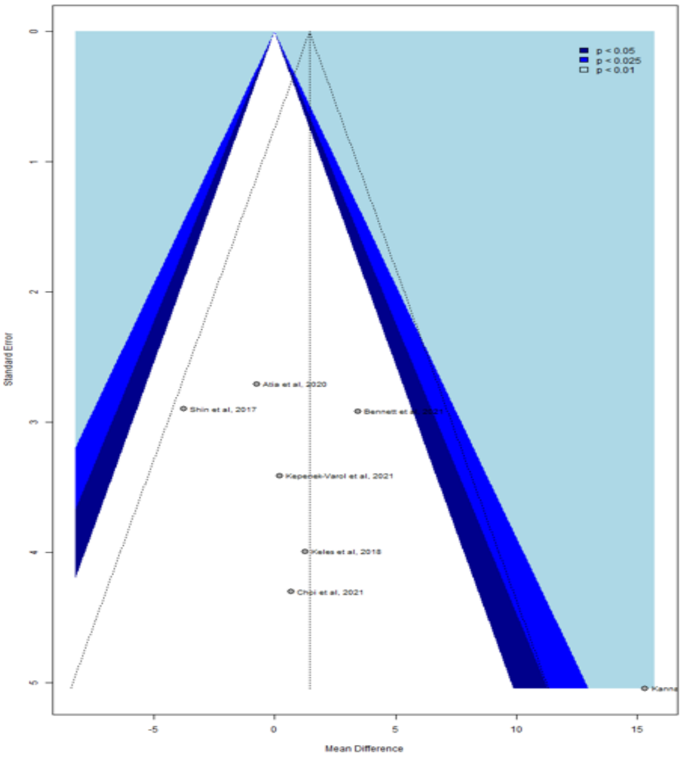

Figure S15. Funnel plot of studies with FVC a.) in litre and b.) in percentage outcome after supplementary respiratory therapy plus conventional care versus conventional care alone [22,23,24,20,21,25,32,26,28,31]

a)

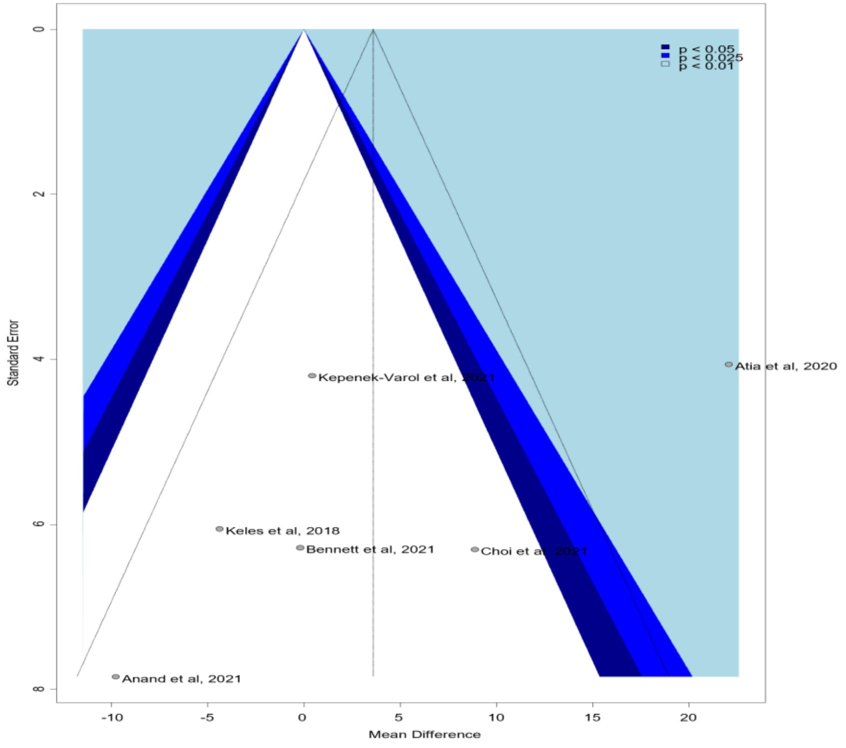

b)

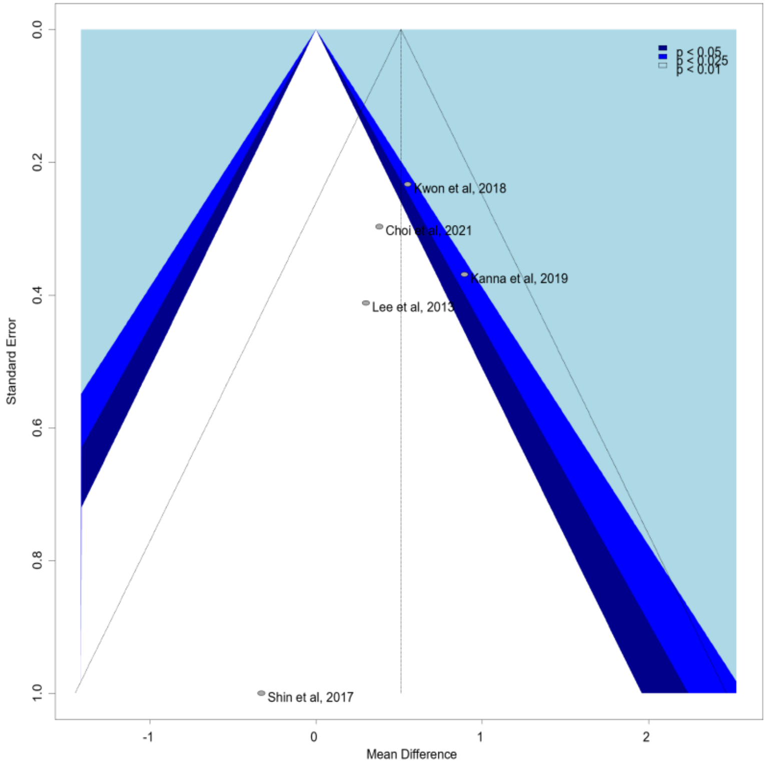

Figure S16. Funnel plot of studies with FEV1 a.) in litre and b.) in percentage outcome after supplementary respiratory therapy plus conventional care versus conventional care alone [27,23,22,20,24,21,25,28,26,31,32]

a)

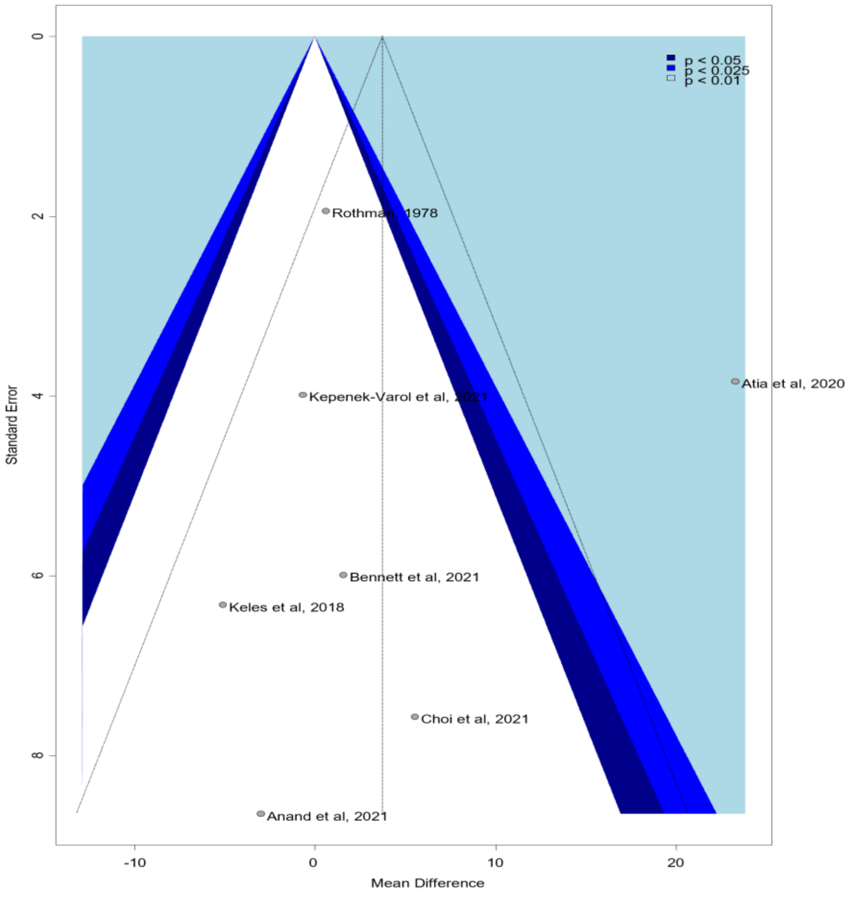

b)

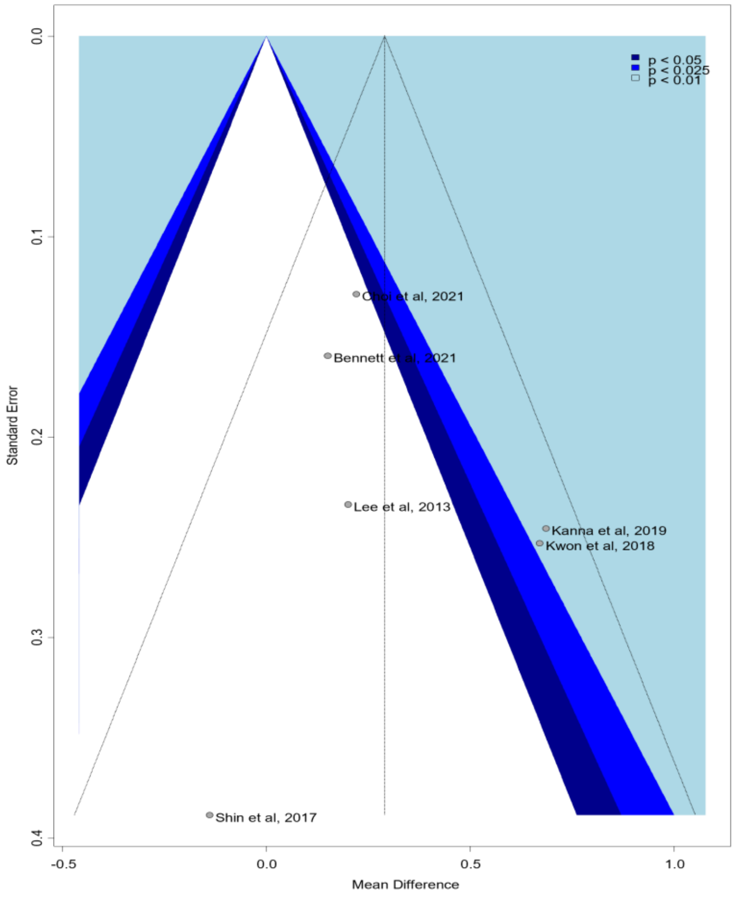

Figure S17. Funnel plot of studies with PEF a.) in litre and b.) PEF in percentage outcome after supplementary respiratory therapy plus conventional care versus conventional care alone [32,21,26,28,31, 23,22,24,25]

a)

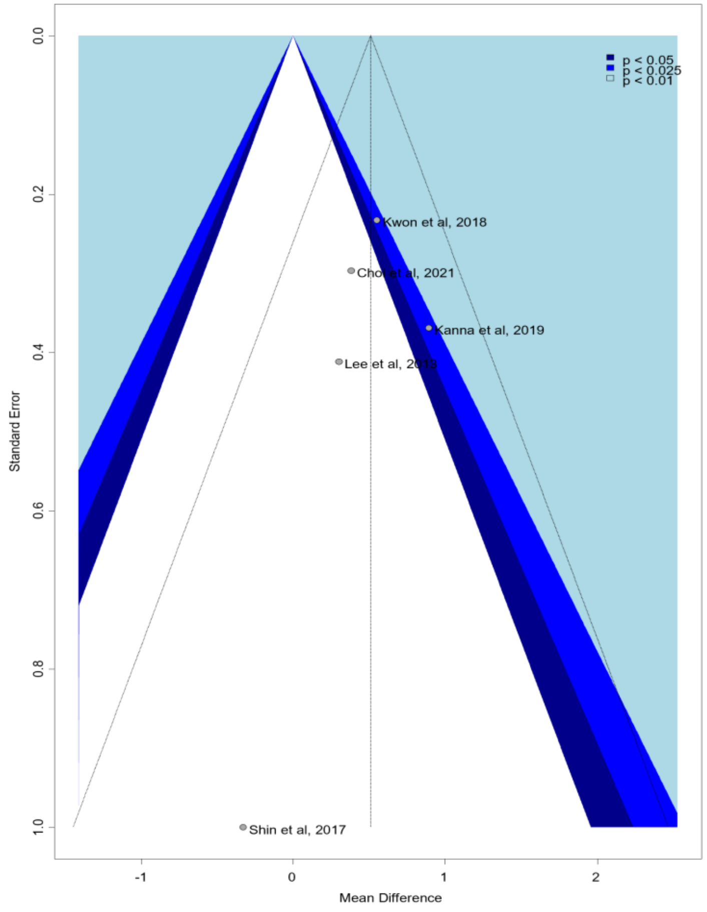

b)

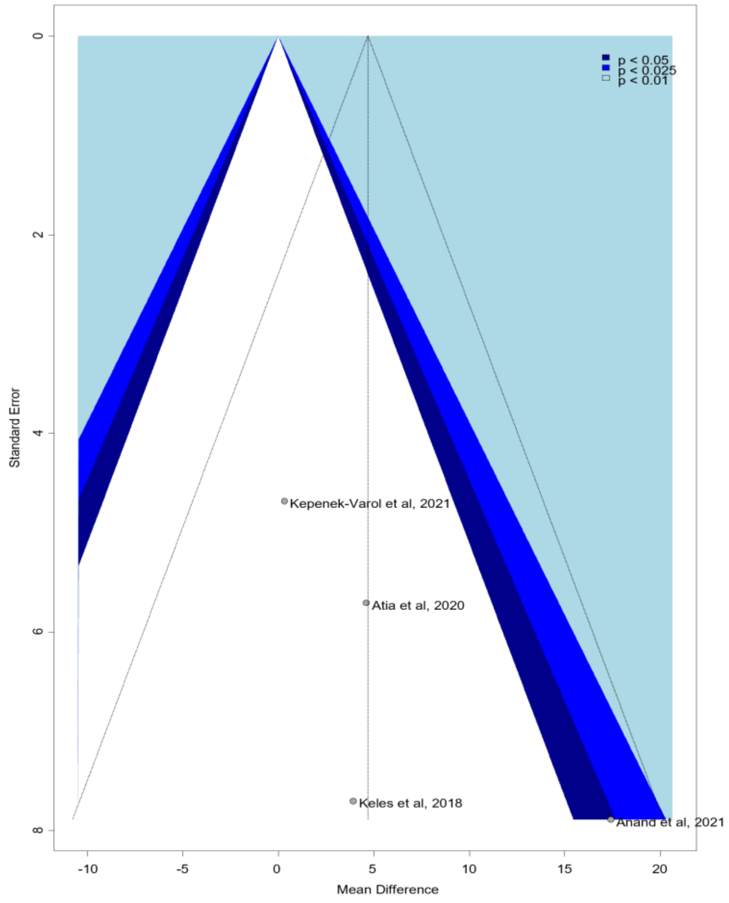

Figure S18. Risk of bias assessment (Rob2 tool)[6] *Outcome: pulmonary functions and respiratory muscle strength [25,22,20,21,29,26,24,23,32,28,27,32]*

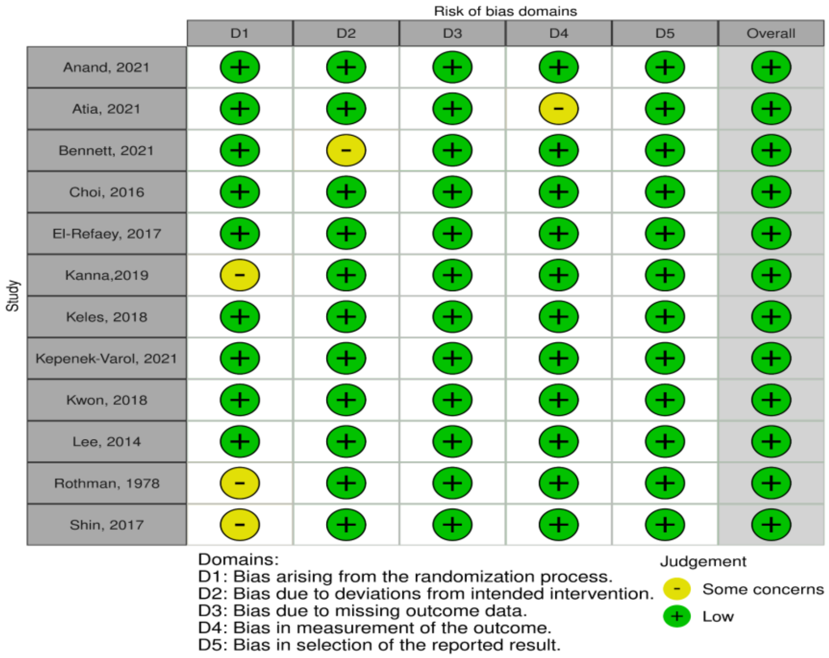

a)

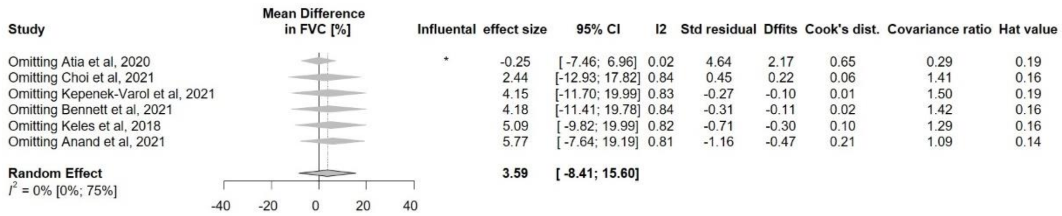

b)

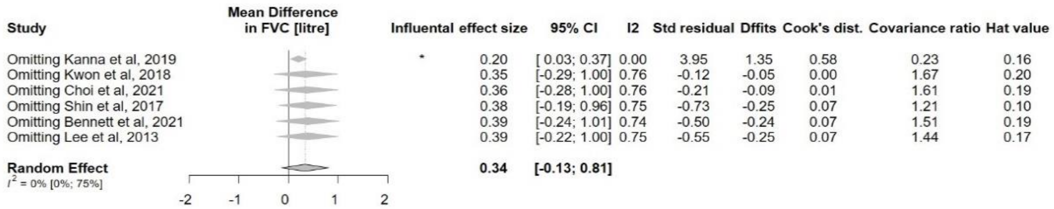

Figure S19. Leave-one-out analyses, sorted by effect size plots of studies with FVC outcomes.(a) in percentage (b) in liter. Explanations oft the plots are in 'Detailed description of synthesis methods' [22,21,23,20,24,25,26,32,21,31,28]

a)

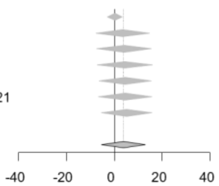

Study	Mean Difference in FEV1 [%]	Influental	effect size	95% CI	I2	Std residual	Dffits	Cook's dist.	Covariance ratio	Hat value
Omitting Atia et al, 2020		*	0.22	[-2.83; 3.27]	0.00	5.57	2.25	0.79	0.15	0.17
Omitting Choi et al, 2021			3.43	[-7.72; 14.59]	0.85	0.16	0.07	0.01	1.29	0.11
Omitting Bennett et al, 2021			4.01	[-7.25; 15.27]	0.85	-0.20	-0.06	0.00	1.34	0.14
Omitting Rothman, 1978			4.34	[-7.11; 15.79]	0.82	-0.34	-0.14	0.02	1.44	0.19
Omitting Anand et al, 2021			4.48	[-6.29; 15.25]	0.84	-0.56	-0.18	0.04	1.20	0.10
Omitting Kepenek-Varol et al, 2021			4.52	[-6.71; 15.74]	0.84	-0.45	-0.18	0.04	1.36	0.16
Omitting Keles et al, 2018			5.06	[-5.42; 15.55]	0.84	-0.86	-0.33	0.11	1.18	0.13
**Random Effect**			**3.74**	**[-5.43; 12.91]**						
I^2 = 0% [0%; 71%]										

b)

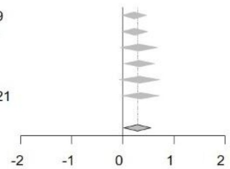

Study	Mean Difference in FEV1 [liter]	Influental	effect size	95% CI	I2	Std residual	Dffits	Cook's dist.	Covariance ratio	Hat value
Omitting Kanna et al, 2019			0.23	[-0.01; 0.47]	0.05	1.75	0.69	0.41	0.83	0.12
Omitting Kwon et al, 2018			0.24	[-0.02; 0.49]	0.13	1.63	0.63	0.35	0.83	0.12
Omitting Lee et al, 2013			0.32	[-0.05; 0.69]	0.44	-0.38	-0.28	0.10	1.74	0.13
Omitting Shin et al, 2017			0.32	[0.02; 0.62]	0.34	-1.10	-0.33	0.11	1.21	0.05
Omitting Choi et al, 2021			0.33	[-0.06; 0.73]	0.42	-0.42	-0.33	0.22	2.26	0.33
Omitting Bennett et al, 2021			0.35	[-0.02; 0.71]	0.37	-0.77	-0.50	0.36	1.78	0.25
**Random Effect**			**0.29**	**[0.02; 0.56]**						
I^2 = 0% [0%; 75%]										

Figure S20. Leave-one-out analyses, sorted by effect size plots of studies with FEV1 outcomes.(a) in percentage (b) in liter. Explanations oft the plots are in 'Detailed description of synthesis methods' [22,21,20,27,25,23,24,26,32,28,31]

a)

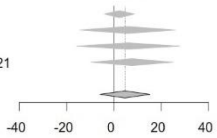

Study	Mean Difference in PEF [%]	Influental	effect size	95% CI	I2	Std residual	Dffits	Cook's dist.	Covariance ratio	Hat value
Omitting Anand et al, 2021		*	2.38	[-4.04; 8.80]	0.00	1.76	0.75	0.55	1.09	0.15
Omitting Keles et al, 2018			5.65	[-14.47; 25.76]	0.42	-0.17	-0.26	0.09	1.95	0.16
Omitting Atia et al, 2020			5.83	[-15.80; 27.46]	0.43	-0.13	-0.26	0.13	2.57	0.28
Omitting Kepenek-Varol et al, 2021		*	7.65	[-9.61; 24.91]	0.02	-1.20	-0.99	0.89	1.60	0.41
**Random Effect**			**4.70**	**[-5.78; 15.17]**						
I^2 = 0% [0%; 85%]										

b)

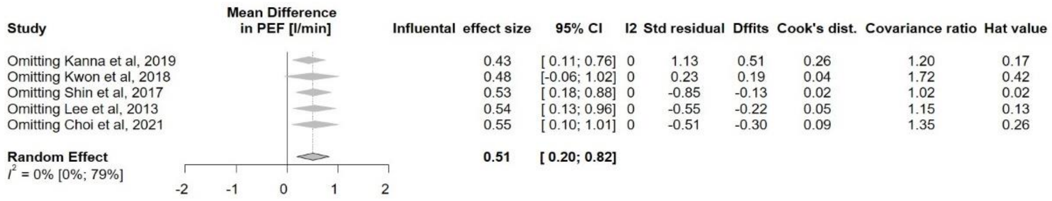

Study	Influential	effect size	95% CI	I2	Std residual	Dffits	Cook's dist.	Covariance ratio	Hat value
Omitting Kanna et al, 2019		0.43	[0.11; 0.76]	0	1.13	0.51	0.26	1.20	0.17
Omitting Kwon et al, 2018		0.48	[-0.06; 1.02]	0	0.23	0.19	0.04	1.72	0.42
Omitting Shin et al, 2017		0.53	[0.18; 0.88]	0	-0.85	-0.13	0.02	1.02	0.02
Omitting Lee et al, 2013		0.54	[0.13; 0.96]	0	-0.55	-0.22	0.05	1.15	0.13
Omitting Choi et al, 2021		0.55	[0.10; 1.01]	0	-0.51	-0.30	0.09	1.35	0.26
**Random Effect**		**0.51**	**[0.20; 0.82]**						
I^2 = 0% [0%; 79%]									

Figure S21. Leave-one-out analyses, sorted by effect size plots of studies with PEF outcomes.(a) in percentage (b) in liter. Explanations oft the plots are in 'Detailed description of synthesis methods' [25,24,22,23,26,32,31,28,21]

a)

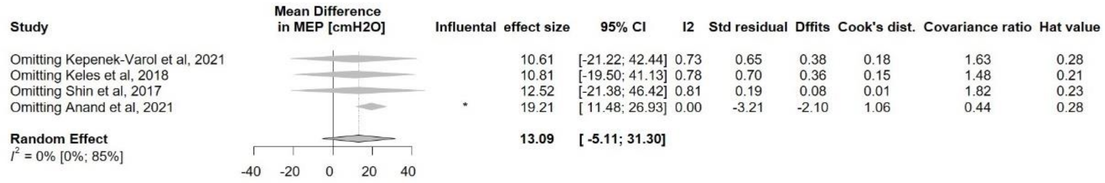

Study	Influential	effect size	95% CI	I2	Std residual	Dffits	Cook's dist.	Covariance ratio	Hat value
Omitting Kepenek-Varol et al, 2021		10.61	[-21.22; 42.44]	0.73	0.65	0.38	0.18	1.63	0.28
Omitting Keles et al, 2018		10.81	[-19.50; 41.13]	0.78	0.70	0.36	0.15	1.48	0.21
Omitting Shin et al, 2017		12.52	[-21.38; 46.42]	0.81	0.19	0.08	0.01	1.82	0.23
Omitting Anand et al, 2021	*	19.21	[11.48; 26.93]	0.00	-3.21	-2.10	1.06	0.44	0.28
**Random Effect**		**13.09**	**[-5.11; 31.30]**						
I^2 = 0% [0%; 85%]									

b)

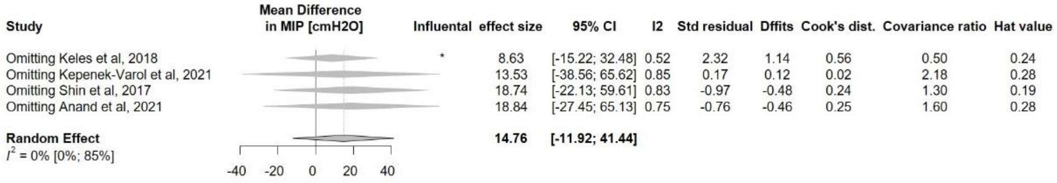

Study	Influential	effect size	95% CI	I2	Std residual	Dffits	Cook's dist.	Covariance ratio	Hat value
Omitting Keles et al, 2018	*	8.63	[-15.22; 32.48]	0.52	2.32	1.14	0.56	0.50	0.24
Omitting Kepenek-Varol et al, 2021		13.53	[-38.56; 65.62]	0.85	0.17	0.12	0.02	2.18	0.28
Omitting Shin et al, 2017		18.74	[-22.13; 59.61]	0.83	-0.97	-0.48	0.24	1.30	0.19
Omitting Anand et al, 2021		18.84	[-27.45; 65.13]	0.75	-0.76	-0.46	0.25	1.60	0.28
**Random Effect**		**14.76**	**[-11.92; 41.44]**						
I^2 = 0% [0%; 85%]									

Figure S22. Leave-one-out analyses, sorted by effect size plots of studies with RMS outcomes.(a) in MEP (b) in MIP. Explanations oft the plots are in 'Detailed description of synthesis methods' [23,24,31,25]

Table S4. PRISMA 2020 Checklist[7]

Section and Topic	Item #	Checklist item	Location where item is reported
**TITLE**			
Title	1	Identify the report as a systematic review.	page 1
**ABSTRACT**			
Abstract	2	See the PRISMA 2020 for Abstracts checklist.	page 2
**INTRODUCTION**			
Rationale	3	Describe the rationale for the review in the context of existing knowledge.	page 5
Objectives	4	Provide an explicit statement of the objective(s) or question(s) the review addresses.	page 5
**METHODS**			
Eligibility criteria	5	Specify the inclusion and exclusion criteria for the review and how studies were grouped for the syntheses.	page 6
Information sources	6	Specify all databases, registers, websites, organisations, reference lists and other sources searched or consulted to identify studies. Specify the date when each source was last searched or consulted.	page 5
Search strategy	7	Present the full search strategies for all databases, registers and websites, including any filters and limits used.	page 6, Table 2S
Selection process	8	Specify the methods used to decide whether a study met the inclusion criteria of the review, including how many reviewers screened each record and each report retrieved, whether they worked independently, and if applicable, details of automation tools used in the process.	page 6
Data collection process	9	Specify the methods used to collect data from reports, including how many reviewers collected data from each report, whether they worked independently, any processes for obtaining or confirming data from study investigators, and if applicable, details of automation tools used in the process.	page 6
Data items	10a	List and define all outcomes for which data were sought. Specify whether all results that were compatible with each outcome domain in each study were sought (e.g. for all measures, time points, analyses), and if not, the methods used to decide which results to collect.	page 6
	10b	List and define all other variables for which data were sought (e.g. participant and intervention characteristics, funding sources). Describe any assumptions made about any missing or unclear information.	pages 6
Study risk of bias assessment	11	Specify the methods used to assess risk of bias in the included studies, including details of the tool(s) used, how many reviewers assessed each study and whether they worked independently, and if applicable, details of automation tools used in the process.	page 6
Effect measures	12	Specify for each outcome the effect measure(s) (e.g. risk ratio, mean difference) used in the synthesis or presentation of results.	pages 7<br>Suppl. material (page 4-5)
Synthesis methods	13a	Describe the processes used to decide which studies were eligible for each synthesis (e.g. tabulating the study intervention characteristics and comparing against the planned groups for each synthesis (item #5)).	page 6
	13b	Describe any methods required to prepare the data for presentation or synthesis, such as handling of missing summary statistics, or data conversions.	page 6<br>Suppl. material (page 4-5)

Section and Topic	Item #	Checklist item	Location where item is reported
	13c	Describe any methods used to tabulate or visually display results of individual studies and syntheses.	pages 6 Suppl. material (pages 5-6)
	13d	Describe any methods used to synthesize results and provide a rationale for the choice(s). If meta-analysis was performed, describe the model(s), method(s) to identify the presence and extent of statistical heterogeneity, and software package(s) used.	page 6 Suppl. material (pages 5-6)
	13e	Describe any methods used to explore possible causes of heterogeneity among study results (e.g. subgroup analysis, meta-regression).	page 6
	13f	Describe any sensitivity analyses conducted to assess robustness of the synthesized results.	Suppl. material (pages 4-5)
Reporting bias assessment	14	Describe any methods used to assess risk of bias due to missing results in a synthesis (arising from reporting biases).	Figures 14S-17S
Certainty assessment	15	Describe any methods used to assess certainty (or confidence) in the body of evidence for an outcome.	page 6
**RESULTS**			
Study selection	16a	Describe the results of the search and selection process, from the number of records identified in the search to the number of studies included in the review, ideally using a flow diagram.	page 8, Figure 1
	16b	Cite studies that might appear to meet the inclusion criteria, but which were excluded, and explain why they were excluded.	page 11,
Study characteristics	17	Cite each included study and present its characteristics.	Table 4S
Risk of bias in studies	18	Present assessments of risk of bias for each included study.	Figure 4S
esults of individual studies	19	For all outcomes, present, for each study: (a) summary statistics for each group (where appropriate) and (b) an effect estimates and its precision (e.g. confidence/credible interval), ideally using structured tables or plots.	pages 8-11, Figures 2-9 Figures 1S-3S
Results of syntheses	20a	For each synthesis, briefly summarise the characteristics and risk of bias among contributing studies.	
	20b	Present results of all statistical syntheses conducted. If meta-analysis was done, present for each the summary estimate and its precision (e.g. confidence/credible interval) and measures of statistical heterogeneity. If comparing groups, describe the direction of the effect.	pages 8-11, Figures 2a-9b Figures 1S-12S
	20c	Present results of all investigations of possible causes of heterogeneity among study results.	page 6, page 13
	20d	Present results of all sensitivity analyses conducted to assess the robustness of the synthesized results.	Not shown
Reporting biases	21	Present assessments of risk of bias due to missing results (arising from reporting biases) for each synthesis assessed.	NA

Section and Topic	Item #	Checklist item	Location where item is reported
Certainty of evidence	22	Present assessments of certainty (or confidence) in the body of evidence for each outcome assessed.	Figures 2a-9b, Figures 1S-12S
**DISCUSSION**			
Discussion	23a	Provide a general interpretation of the results in the context of other evidence.	page 12-13
	23b	Discuss any limitations of the evidence included in the review.	page 13
	23c	Discuss any limitations of the review processes used.	page 13
	23d	Discuss the implications of the results for practice, policy, and future research.	pages 13-14
**OTHER INFORMATION**			
Registration and protocol	24a	Provide registration information for the review, including the register name and registration number, or state that the review was not registered.	page 5
	24b	Indicate where the review protocol can be accessed, or state that a protocol was not prepared.	page 5
	24c	Describe and explain any amendments to the information provided at registration or in the protocol.	page 5
Support	25	Describe sources of financial or non-financial support for the review, and the role of the funders or sponsors in the review.	page 3
Competing interests	26	Declare any competing interests of review authors.	page 3
Availability of data, code and other materials	27	The report which of the following are publicly available and where they can be found: template data collection forms; data extracted from included studies; data used for all analyses; analytic code; any other materials used in the review.	pages 6-7, Suppl. material

Table S2. Search key in the databases (MEDLINE, Embase, CENTRAL, Web of Science and Scopus)

'cerebral palsy' OR 'CP' OR 'spastic paraplegia' OR 'cerebral paresis' OR dipleg OR 'spastic OR hemipleg*OR 'spastic quadripleg*' OR 'Little's Disease
'breathing exercises' OR 'breathing therapy' OR 'pulmonary therapy, OR 'respiratory therapy' OR 'respiratory measurement, OR 'respiratory muscles' OR 'inspiratory training, OR 'inspiratory therapy' OR 'physical therapy

Table S3. Summary of findings: Grading of Recommendations, Assessment, Development and Evaluations (GRADE) framework

Certainty assessment							№ of patients		Effect		Certainty	Importance
№ of studies	Study design	Risk of bias	Inconsistency	Indirectness	Imprecision	Other considerations	supplementary respiratory therapy	conventional care	Relative (95% CI)	Absolute (95% CI)		
**pulmonary functions (FVC)_in Liter (assessed with spirometer)**												
6	randomized trials	not serious	serious	not serious	not serious	none	101	99	-	**0** (0.13 lower to 0.81 higher)	⨁⨁⨁◯ Moderate	IMPORTANT
**pulmonary function (FVC)_in % (assessed with spirometer)**												
6	randomized trials	not serious	serious	not serious	not serious	none	78	68	-	**0** (8.41 lower to 15.6 higher)	⨁⨁⨁◯ Moderate	IMPORTANT
**pulmonary function FEV1_in Liter (assessed with: spirometry)**												
6	randomized trials	not serious	not serious	not serious	not serious	none	106	104	-	**0** (0.02 lower to 0.56 higher)	⨁⨁⨁⨁ High	IMPORTANT
**pulmonary function FEV1_in % (assessed with: spirometry)**												
7	randomized trials	not serious	serious	not serious	not serious	none	78	67	-	**0** (5.43 lower to 12.91 higher)	⨁⨁⨁◯ Moderate	IMPORTANT
**pulmonary function FVC/FEV1 % (assessed with: spirometry)**												
7	randomized trials	not serious	not serious	not serious	not serious	none	133	118	-	**0** (3.43 lower to 6.32 higher)	⨁⨁⨁⨁ High	IMPORTANT
**pulmonary function PEF in Liter (assessed with: spirometry)**												
5	randomized trials	not serious	not serious	not serious	not serious	none	73	70	-	**0** (0.2 higher to 0.82 higher)	⨁⨁⨁⨁ High	IMPORTANT
**pulmonary function PEF in % (assessed with: spirometry)**												
4	randomized trials	not serious	not serious	not serious	serious	none	46	50	-	**0** (5.78 lower to 15.17 higher)	⨁⨁⨁◯ Moderate	IMPORTANT

Certainty assessment							№ of patients		Effect		Certainty	Importance
№ of studies	Study design	Risk of bias	Inconsistency	Indirectness	Imprecision	Other considerations	supplementary respiratory therapy	conventional care	Relative (95% CI)	Absolute (95% CI)		
**Respiratory muscle strength MEP (maximal expiratory pressure) (assessed with: manovacumetry)**												
4	randomised trials	not serious	serious	not serious	serious	none	56	54	-	**0** (5.11 lower to 31.3 higher)	⨁⨁◯◯ Low	IMPORTANT
**Respiratory muscle strength MIP (maximal inspiratory pressure) (assessed with: manovacumetry)**												
4	randomised trials	not serious	serious	not serious	serious	none	56	54	-	**0** (11.92 lower to 41.44 higher)	⨁⨁◯◯ Low	IMPORTANT

CI: confidence interval

Explanations

a. Clinically heterogeneous population
b. Small sample size, wide CI

Table S1. Baseline characteristics of the included RCTs

Study	Study Design	Country	Intervention	Comparator	Year Range	GMFCS level	Frequency of intervention (per week)	Duration of intervention (Min)	Length of intervention	Outcomes
Anand et al., 2021	RCT	India	inspiratory muscle training	sensorimotor exercises	8-15	I-III	3	15	6 weeks	FVC, FEV1, FVC/FEV1%, PEF
Atia and Tharwat, 2021	RCT	Egypt	incentive spirometer	traditional physiotherapy	8-12	II-IV	3	15	8 weeks	FVC, FEV1, PEF, MIP, MEP
Bennett et al., 2021	RCT	Thailand	manual diaphragmatic stretching technique	standard physiotherapy	8-18	I-IV	3	10	6 weeks	FVC, FEV1, FEV1/FVC%
Choi et al.,2016	RCT	Korea	incentive spirometer	comprehensive rehabilitation therapy	8-15	I-IV	2	NA	4 weeks	FVC, FEV1, PEF
Keles et al, 2018	RCT	Turkey	inspiratory muscle training at 30% of MIP	conventional rehabilitation + sham (5% of MIP)	7-14	I-II	7	30	6 weeks	FVC, FEV1, FEV1/FVC%, PEF, MIP, MEP, PEDI-FSS, PEDI-CAS, CPQOL-child,
Kepenek-Varol et al., 2021	RCT	Turkey	inspiratory muscle training	conventional physiotherapy rehabilitation program	7-16	I-II (only hemiplegic)	7	15	8 weeks	FVC, FEV1, FEV1/FVC, PEF, MIP, MEP
Lee at al. 2013	RCT	Korea	feedback respiratory training	comprehensive rehabilitation	6-12	NA	3	15	4 weeks	FVC, FEV1, PEF

Kanna and Balabaskar, 2019	RCT	India	breathing exercises	neurodevelopmental therapy	NA	NA*(only spastic quadriplegic)*	5	15	6 weeks	FVC, FEV1, FVC/FEV1%, PEF
Rothman, 1978	RCT	Israel	breathing exercises	conventional care	5-10	NA	7	5-7	8 weeks	VC, FEV1
Shin and Kim 2017	RCT	Korea	upper extremity resistance exercise with elastic bands	conventional care for 30 min.	9.25+-3.65	I-III	2	20-30	8 weeks	FVC, FEV1, PEF, MIP, MEP
El Refaey et al. 2017	RCT	Egypt	feedback respiratory training	conventional physical therapy program	6-12	III-IV	5	2*15	4 weeks	MIP, MEP, PedsQL
Kwon and Kim, 2018	RCT	Korea	resistance exercises	conventional	5-12	II-III	2	40	12 weeks	FVC, FEV1, PEF
Litscke et al., 2012*	RCT	USA, Texas	concurrent flow resistance	concurrent pressure threshold device	NA	athletes	NA	NA	9 weeks	HRQoL

FVC forced vital capacity; FEV1 forced expiratory volume in 1 second, VC vital capacity, PEF peak expiratory flow, MIP maximal expiratory volume, MEP maximal expiratory volume, PedSQL Pediatric Quality of Life Inventory, PEDI FSS Pediatric Evaluation of Disability Inventory Functional Skills Scale, PEDI CAS Pediatric Evaluation of Disability Inventory Caregiver Assistant Scale, CP QOL- Child Cerebral Palsy Quality of Life Questionnaire for Children

*not included in the meta-analysis

~~References~~

1. IntHout J, Ioannidis JP, Borm GF. The Hartung-Knapp-Sidik-Jonkman method for random effects meta-analysis is straightforward and considerably outperforms the standard DerSimonian-Laird method. *BMC Med Res Methodol* 2014; **14**: 25.
2. Knapp G, Hartung J. Improved tests for a random effects meta-regression with a single covariate. *Stat Med* 2003; **22**: 2693–710.
3. Jackson D, Turner R. Power analysis for random-effects meta-analysis. *Res Synth Methods* 2017; **8**: 290–302.
4. Harrer M, Cuijpers P, Furukawa TA, Ebert DD. Doing Meta-Analysis with R. Boca Raton: Chapman and Hall/CRC, 2021 DOI:10.1201/9781003107347.
5. Veroniki AA, Jackson D, Viechtbauer W, et al. Methods to estimate the between-study variance and its uncertainty in meta-analysis. *Res Synth Methods* 2016; **7**: 55–79.
6. McGuinness LA, Higgins JPT. Risk-of-bias VISualization (robvis): An R package and Shiny web app for visualizing risk-of-bias assessments. *Res Synth Methods* 2021; **12**: 55–61.
7. Page MJ, McKenzie JE, Bossuyt PM, et al. The PRISMA 2020 statement: an updated guideline for reporting systematic reviews. *BMJ* 2021; **372**: n71.
8. Anand B, Karthikbabu S. Effects of additional inspiratory muscle training on mobility capacity and respiratory strength for school-children and adolescents with cerebral palsy: a randomized controlled trial. *Braz J Phys Ther* 2021; **25**: 891–9.
9. Atia DT, Tharwat MM. Effect of incentive spirometer exercise combined with physical therapy on pulmonary functions in children with cerebral palsy. *Int J Ther Rehabil* 2021; **28**. DOI:10.12968/ijtr.2020.0072.
10. Bennett S, Siritaratiwat W, Tanrangka N, Bennett MJ, Kanpittaya J. Effectiveness of the manual diaphragmatic stretching technique on respiratory function in cerebral palsy: A randomised controlled trial. *Respir Med* 2021; **184**. DOI:10.1016/j.rmed.2021.106443.
11. Choi JY, Rha DW, Park ES. Change in pulmonary function after incentive spirometer exercise in children with spastic cerebral palsy: A randomized controlled study. *Yonsei Med J* 2016; **57**: 769–75.
12. Keles MN, Elbasan B, Apaydin U, Aribas Z, Bakirtas A, Kokturk N. Effects of inspiratory muscle training in children with cerebral palsy: a randomized controlled trial. *Braz J Phys Ther* 2018; **22**: 493–501.
13. Kepenek-Varol B, Gürses HN, İçağasıoğlu DF. Effects of Inspiratory Muscle and Balance Training in Children with Hemiplegic Cerebral Palsy: A Randomized Controlled Trial. *Dev Neurorehabil* 2022; **25**: 1–9.
14. Lee HY, Cha YJ, Kim K. The effect of feedback respiratory training on pulmonary function of children with cerebral palsy: a randomized controlled preliminary report. *Clin Rehabil* 2014; **28**: 965–71.
15. Santhosh Kanna BS, Balabaskar K. A study on efficacy of respiratory exercises coupled with neuro developmental treatment on pulmonary function of children with spastic quadriplegic cerebral palsy. *Biomedical and Pharmacology Journal* 2019; **12**: 1519–24.
16. Rothman JG. Effects of Respiratory Exercises on the Vital Capacity and Forced Expiratory Volume in Children with Cerebral Palsy. *Phys Ther* 1978; **58**: 421–5.
17. Shin S-O, Kim N-S. Upper extremity resistance exercise with elastic bands for respiratory function in children with cerebral palsy. .

18. Hamdy El-Refaey B, Mosaad G, Maksoud A-E, Ali I. Efficacy of feedback respiratory training on respiratory muscle strength and quality of life in children with spastic cerebral palsy: Randomized controlled trial. *Bulletin of Faculty of Physical Therapy* 2017; **22**: 46–52.
19. Kwon H-Y, Kim B-J. Effects of task-specific movement patterns during resistance exercise on the respiratory functions and thickness of abdominal muscles of children with cerebral palsy: randomized placebo-controlled double-blinded clinical trial. *J Phys Ther Sci* 2018; **30**: 1073–80.
20. Litchke L, Lloyd L, Schmidt E, Russian C, Reardon R. Effects of Concurrent Respiratory Resistance Training on Health-Related Quality of Life in Wheelchair Rugby Athletes: A Pilot Study. *Top Spinal Cord Inj Rehabil* 2012; **18**: 264–72.
